# Optimization of signalized network configurations using the Lane-based method

**DOI:** 10.1371/journal.pone.0216958

**Published:** 2019-06-10

**Authors:** C. K. Wong, Yi Liu

**Affiliations:** Department of Architecture and Civil Engineering, City University of Hong Kong, Kowloon Tong, Hong Kong SAR; Huazhong University of Science and Technology, CHINA

## Abstract

Vehicle movements at signalized intersections should follow the guidance of lane marking arrows. Turns are permitted or banned depending on the existence of lane marking arrows establishing network link connectivity. Lane markings are the interface that joins consecutive upstream and downstream intersections. Traffic flows from origins to destinations across intersections should be governed by lane markings. In this study, conventionally fixed lane markings are relaxed as binary variables to be optimized by maximizing the green bandwidths. The proposed methodology is innovative in that it extends the lane-based design framework to incorporate green band maximization for enhancing traffic signal coordination. Path flows are controlled to satisfy flow conservations and to compile turning flows at intersections. With these turning flows as inputs, lane markings can be optimized together with the coordinated traffic signal settings. Path flows, path travel times, and path choices are evaluated through new linear constraints. For path travel times, cruise times along lanes and total delays at the ends of lanes are evaluated. The non-linear total delays are approximated by the proposed linearized delay function. The model coefficients are calibrated by network data as in a previous study. The problem is formulated as a binary-mixed-integer-linear-program and solved by standard branch-and-bound techniques using a CPLEX solver. To avoid non-linearity in the problem formulation, the bandwidth maximization approach is adopted instead of minimizing the total network delay in the design of the signalized network. A network with four intersections is provided to demonstrate how optimized lane markings can create efficient network link configurations. The numerical results are promising compared with those obtained in previous studies and show that the overall network performance can be improved.

## Introduction

In road networks, links with shorter travel times are preferred by users over travel between origins and destinations (OD). Route choices depend on total travel times along links and delays at intersections. In signalized networks, signal settings can affect route choices [1–5]. Examples of routing optimization problems for moving hazardous materials are based on multi-objective functions by probabilistic search methods such as those of neural networks and genetic algorithms [6–9]. Coordinated signal controls in ring road systems can be improved by proper speed guidance [10]. Network flow patterns depend on signal settings that are updated until consistent patterns are achieved [11–13]. A comprehensive review of this approach can be found in the literature [14]. A bi-level programing formulation integrated a lower-level user equilibrium assignment model and an upper-level control optimization model. Signal operations were optimized in the upper level and users’ equilibrium was achieved in the lower level [15–20].

The coordination of linked traffic signals is important when considering signalized networks. The TRANSYT traffic model simulates network traffic considering platoon dispersions and signal coordination [21]. A path-based assignment routine was developed to enhance the TRANSYT model so that traffic flows could become responsive to signal settings [22]. Group-based signal optimization was conducted for this enhanced TRANSYT platform [23]. A sensitivity analysis, based on the derivatives of the performance index with respect to signal settings, was conducted to optimize the network signal settings [24]. By displaying different signal timings, shorter or longer paths inside networks are generated as OD route choices for users. Linked connections also determine the availability of those paths. Such network configurations have normally been given as users’ fixed inputs. However, lane marking arrows show users which turns are permitted when approaching intersection levels are variables and should be designed according to the demand flow patterns [25–28]. Practical demonstrations of the lane-based methods for signalized intersections can be found in the literature [29, 30]. More recent studies applying the concept of lane-based design for signalized intersections are noted below. The lane assignments for multiple left-turn and right-turn lanes were determined by optimizing a multi-objective function with microscopic traffic simulations. Transportation efficiency, energy consumption, and road safety were remarkably improved [31]. The lane-based concept, varying the approach lanes at signalized intersections, was used in Dalian. The designs of time-varying traffic signals and intersection settings at different times of the day were produced by minimizing the total delay. Four scenarios with field data verifications demonstrated promising results [32]. Lane allocations and signal timings were optimized to design symmetric intersection layouts. A binary-mixed-integer-linear-program was formulated and solved by the standard branch-and-bound method. Instead of the usual four phases of traffic signal settings at intersections, three were found to be sufficient for separating all conflicting movements [33]. A new stage-based modeling approach was developed to prohibit left-turn traffic in urban road networks by minimizing total travel time [34]. The underlying concept is similar to the one proposed in this study. The proposed lane-based optimization framework is more computationally attractive, generating lane markings that configure an urban road network in which the proper locations to permit or prohibit turns may be optimized. A modeling interface was established between the connectivity of network links and lane marking designs at intersections [35]. Maximizing the common flow multiplier to scale the whole OD (input) demand flow matrix, as done in a previous study [36], can maximize the capacities of all individual signalized intersections within a network and can generate lane markings for configuring the network connections [37]. However, equilibrium flow conditions and proper signal coordination were not considered.

The MAXBAND method was developed to maximize the bandwidths across adjacent intersections and ensure smooth arterial progression across intersections [38]. This concept was extended to address multi-arterial systems [39]. Systematic traffic-dependent criteria were developed for optimizing a weighted sum of bandwidths to form a new MULTIBAND model [40, 41]. MULTIBAND was applied to design asymmetrical traffic signal coordination for arterials [42] and model arterial networks [43]. In the MULTIBAND model, individual bandwidths are weighted based on ratios of flow volumes to saturation flow rates (i.e. flow factors). In their formulation, flow volumes and saturation flow rates are known as given parameters. In the proposed lane-based optimization framework, both flow factors and bandwidths are variables and their products in MULTIBAND become a nonlinear term. Vehicles from the upstream intersections enter downstream links and take the link travel times to the downstream intersections. Average travel times depend on both the physical separation between upstream and downstream intersections and average vehicle speeds. The slopes of the vehicle trajectories in the time-space diagram represent average vehicle speeds [38]. Ideally, vehicles can move without waiting if proper green signal phases and offsets are displayed. Offsets are the time differences between two green start times at the upstream and downstream intersections. The signalized network is well coordinated if the offsets are properly designed.

To optimize the lane marking patterns at individual intersections, 13 sets of linear constraints are required, taking the turning flow patterns as model inputs for defining a feasible solution region for the traffic signal settings [25, 27, 37]. The constraints are set to (i) ensure that the number of lane marking arrows for turns from upstream do not exceed their respective numbers of downstream exit lanes; (ii) satisfy the requirements for flow conservation, equalizing the given turning flows and the sum of assigned lane flows; (iii) establish lane markings to provide consistency in the assigned turning flows; (iv) avoid (internal) conflicts of lane markings across adjacent approach lanes from the same approaches; (v) establish single lane markings (for a single turn) or shared lane markings (for multiple turns) on approach lanes, thus ensuring the use of all approach lanes; (vi) restrict the optimized cycle time to be within the users’ specified allowable range; (vii) ensure that the optimized green duration times exceed the users’ specified minimum duration of green times; (viii) restrain the starts of green times within the traffic signal cycle; (ix) provide minimum clearance times (or intergreen times) to separate the right-of-way of conflicting traffic movements; (x) introduce successor functions to regulate the signal display orders for conflicting pairs of traffic movements; (xi) synchronize the traffic signal settings for turns and lanes according to the optimized lane marking patterns; (xii) equalize the flow factors on adjacent approach lanes with identical lane marking designs on both approach lanes; and (xiii) restrict the maximum degree of saturation to below the users’ specified figure for all approach lanes. These 13 sets of governing constraints are standard formulation for the lane-based design for individual signalized intersections and have been applied for reserve capacity maximization [25], total delay minimization [26], and intersection layouts with short lanes [30]. Other applications can also be found for U-turn traffic control [44], designing the layout of a displaced left-turn intersection [45], handling fluctuating traffic demand [46], urban arterials with reversible lane usages [47], for exclusive bus lanes [48], and designing pre-signals with sorting areas before entering the common areas of intersections [49].

As reviewed above, in most research methods relating to signalized network designs the network configurations are fixed users’ inputs, including the connections of network links and their link saturation flow rates, indicating that their discharge abilities are excluded from the optimization framework. The given OD flows might be forced to use the available but congested paths provided by inefficient link connections leading to sub-optimal flow distributions. In a recent study [37], lane markings were optimized by maximizing the common flow multiplier to the given OD matrix, thus generating network configurations. However, equilibrium flow conditions and the coordination of traffic signal settings were not modeled, leading to unrealistic results. In this study, users’ movement paths are free to be generated depending on the optimization results of lane markings and signal timings that serve all OD flows from the given matrix. This study attempts to relax lane markings as design variables and optimize the network configurations such that the overall link connectivity of a signalized network is enhanced. All network links or lanes can be used effectively to let through all OD demand flows. Offsets and other signal settings are optimized in a unified framework by maximizing the green bandwidths along the selected major routes. Path travel times are evaluated containing lane cruise times and delays at the ends of lanes to present users’ equilibrium flow conditions. A network consisting of four signalized intersections is given as a case study to show the effectiveness of the proposed algorithms. Five scientific measures are evaluated for their assessment of the performance of optimized network settings: (I) the lengths of green bands, (II) the numbers and patterns of lane markings to enable feasible paths for connecting OD pairs, (III) the effectiveness of signal timings to coordinate upstream and downstream intersections, (IV) the equilibrium flow conditions for using different paths to connect the OD pairs, and (V) total network travel times $\left(\sum_{all\;paths}path\;\text{flow}\;\times \;path\;travel\;time\right)$. The mathematical problem is formulated as a binary-mixed-integer-linear-program (BMILP) and is solved by a standard branch-and-bound method through the CPLEX solver. Network settings optimized by the proposed algorithms are then compared with those obtained in a previous study to show their enhancement.

## Methodology

### Notation

$o,o^{\prime }$ Origin node in the signal-controlled network
d Destination node in the signal-controlled network
$T_{o,d}$ Demand flow inputs from origin o to destination d
$h_{o,d}$, ${h^{\prime }}_{o,d}$ Path number from origin o to destination d
${\bar{H}}_{o,d}$ Total number of paths connecting origin o to destination d
$P_{o,d,h_{o,d}}$ Path flow from origin o to destination d using path $h_{o,d}$
O Total number of origin nodes in the signal-controlled network
D Total number of destination nodes in the signal-controlled network
M Arbitrary large positive number
$\alpha_{o,d,h_{o,d}}$ Binary variable to denote the existence of path $h_{o,d}$ connecting origin o to destination d
n,m Intersection number
N Total number of intersections in the signal-controlled network
i,i' Arm number of an intersection
j,j' Arm number of an intersection
$I_{n}$ Total number of arms at intersection n
$Q_{n,i,j}$ Demand turning flow from arm i to arm j at intersection n
F(n,i,j) Mathematical function identifying origin o and destination d along path $h_{o,d}$ that makes a turn from arm i to arm j at intersection n
$\beta_{o,d}$ Binary variable representing demand flow from origin *o* to destination d
k,k' Approach lane number
$L_{n,i}$ Total number of approach lanes from arm i at intersection n
$\delta_{n,i,j,k}$ Binary variable representing the existence of the permitted movement (lane marking) from arm i to arm j on lane k at intersection n (= 1 if it exists or = 0 if it does not exist)
$F^{\prime }(o,d,h_{o,d})$ Mathematical function identifying all turns from arm i to arm j at intersection n for a path $h_{o,d}$ connecting origin o and destination d
$\Delta_{n,i,j,o,d,h_{o,d}}$ Auxiliary binary variable representing the existence of a lane marking permitting a turn from arm i to arm j at intersection *n* using path $h_{o,d}$ to connect origin o and destination d
$\tau_{n,m}$ Average travel time for vehicles leaving from upstream intersection *n* to reach downstream intersection *m*
*S*_*n*,*m*_ Distance between upstream intersection *n* and downstream intersection *m*
${\bar{V}}_{n,m}$ Average travel speed of vehicles between intersection *n* and intersection *m*
$\Theta_{n,i,k}$ Start of green time on lane k from arm i at intersection n
$\Phi_{n,i,k}$ Duration of green time on a lane k from arm i at intersection n
$W_{n,m}$ Green bandwidth between upstream intersection *n* and downstream intersection *m*
$\Lambda_{1,n,i,k,m,i',k'}$ Binary variable stating the Condition I result between a transformed start of green time for vehicle movement of upstream intersection (n,i,j) and the start of green time for vehicle movement of downstream intersection (m,i',k') (= 1 if Condition I holds or = 0 if not)
$\Lambda_{2,n,i,k,m,i',k'}$ Binary variable stating the Condition II result between a transformed end of green time for vehicle movement of upstream intersection (n,i,j) and the end of green time for vehicle movement of downstream intersection (m,i',k') (= 1 if Condition II holds or = 0 if not)
$\Lambda_{3,n,i,k,m,i',k'}$ Binary variable stating the Condition III result between a transformed end of green time for vehicle movement of upstream intersection (n,i,j) and the start of green time for vehicle movement of downstream intersection (m,i',k') (= 1 if Condition III holds or = 0 if not)
$\Lambda_{4,n,i,k,m,i',k'}$ Binary variable stating the Condition IV result between a transformed start of green time for vehicle movement of upstream intersection (n,i,j) and the end of green time for vehicle movement of downstream intersection (m,i',k') (= 1 if Condition IV holds or = 0 if not)
$x_{n,m}$ Number of signal cycle difference for signal phases between upstream intersection *n* and downstream intersection *m* for establishing one continuous green band
D(n,i,j) Mathematical function identifying arm i' at downstream intersection *m* taking traffic from upstream intersection *n* turning from arm i to arm j
${\bar{W}}_{o,d,h_{o,d}}$ Green bandwidth from origin *o* to destination *d* through path $h_{o,d}$
$\Pi_{n,i,k}$ Binary variable stating the lane condition (= 1 when the lane is over-saturated that the degree of saturation ≥1.0 or = 0 when the lane is unsaturated)
$y_{n,i,k}$ Flow factor on lane k from arm i at intersection *n*
$\gamma_{n,i,k}$ Lane travel time on lane k from arm i at intersection *n*
ε Coefficient of flow factor in the link performance function in the unsaturated flow condition
ε' Coefficient of flow factor in the link performance function in the over-saturated flow condition
$\varphi_{n,i,k}$ End of lane delay of lane k from arm i at intersection *n*
$\eta_{c}$ Coefficient of cycle length in the linear approximated delay formula
$\eta_{e}$ Coefficient of effective of green time in the linear approximated delay formula
$\eta_{y}$ Coefficient of flow factor in the linear approximated delay formula
$\eta_{q}$ Coefficient of total lane flow in the linear approximated delay formula
c Cycle length (cycle time in seconds)
ζ Reciprocal of a cycle length (= 1/c)
e Difference between actual and effective green time
$q_{n,i,j,k}$ Lane flow turning from arm i to arm j on lane k at intersection n
$\chi_{o,d,h_{o,d}}$ Path travel time from origin *o* to destination *d* along path $h_{o,d}$
$Κ\left(o,d,h_{o,d}\right)$Mathematical function identifying the traffic movement (n,i,j) along a path $h_{o,d}$ connecting origin o and destination d
$Z_{o,d,h_{o,d}}$ Numerical weight for the importance of the green band from selected OD pairs *o* and *d* through selected path $h_{o,d}$ in the objective function for optimization purposes

### Linear governing constraint sets

#### Flow conservation to equalize path flows and given OD flows

**Fig 1:**
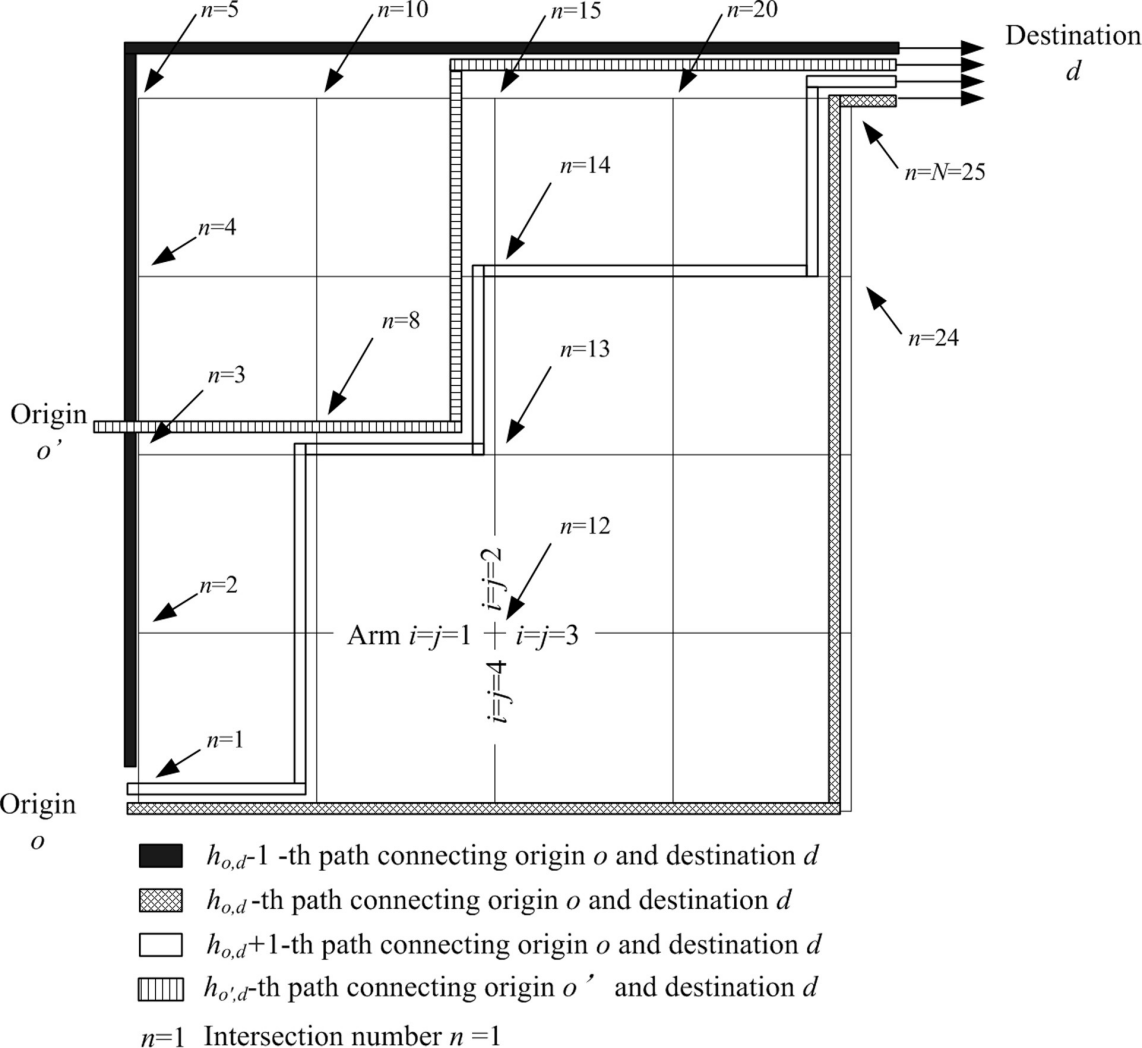
Converting path flows to turning flows at intersections for OD pairs.

Eq (1) is an equality constraint set for equalizing the sum of available path flows and the given OD demand flows, thus satisfying the flow conservation requirements:

$$T_{o,d}=\sum_{h_{o,d}=1}^{{\bar{H}}_{o,d}}P_{o,d,h_{o,d}}\begin{array}{cc} , & \forall o\in \left\{1,2,\ldots ,O\right\},d=\left\{1,2,\ldots ,D\right\} \end{array}$$ (1)

where $T_{o,d}$ is users’ given OD demand flows, o is origin, d is destination, $h_{o,d}$ is a path number for the OD pair, ${\bar{H}}_{o,d}$ is a total number of paths for the OD pair, $P_{o,d,h_{o,d}}$ is a path flow for the OD pair o and d along path $h_{o,d}$, and *O* and *D* are respectively the total numbers of origins and destinations. Fig 1 shows various paths connecting OD pairs o and d and $o^{\prime }$ and d.

#### Path existence for connecting OD pairs

A set of binary variables may be used to denote the path existence for connecting the OD pairs. Eq (2) is developed relate the path flow $P_{o,d,h_{o,d}}$ along path $h_{o,d}$.

$$M^{2}P_{o,d,h_{o,d}}\geq M\alpha_{o,d,h_{o,d}}\geq P_{o,d,h_{o,d}}\begin{array}{cc} , & \forall o\in \left\{1,2,\ldots ,O\right\},d\in \left\{1,2,\ldots ,D\right\},h_{o,d} \end{array}\in \left\{1,2,\ldots ,{\bar{H}}_{o,d}\right\}$$ (2)

where $\alpha_{o,d,h_{o,d}}$ is a binary variable (= 1 if path $h_{o,d}$ exists or = 0 if not) for an OD pair o and d, and M is an arbitrary large numerical figure. Numerically, if $P_{o,d,h_{o,d}}$ = 400 pcu/h, then $\alpha_{o,d,h_{o,d}}$ = 1 representing that path $h_{o,d}$ exists.

#### Turning flows at intersections

OD demand flows are usually given by users as model inputs that are to be assigned onto different paths. Path flows may pass through different intersections in the network. Turning flows from arm *i* to arm *j* at intersection *n* can be deduced from the path flows. Fig 1 shows origin nodes *o* and $o^{\prime }$and destination node *d*. Intersection *n* = 12 is a four-arm intersection, where i=j={1,2,3,4} For (n,i,j)=(12,1,2) or (12,2,3) it stands for a left-turn movement. From origins *o* and $o^{\prime }$, four paths exist to connect the destination *d*. For intersection *n* = 13, from arm *i* = 1 to arm *j* = 2, a left-turn movement lets through the two path flows. Turning flow $Q_{n,i,j}$ at intersection (*n* = 13) should be the sum of path flows $P_{o,d,h_{o,d}+1}$ and $P_{o',d,h_{o',d}}$. For the whole network, Eq (3) is required for compiling all turning flows at the intersection level.

$$Q_{n,i,j}=\sum_{o}^{}\sum_{d}^{}\sum_{h_{o,d}}^{}P_{o,d,h_{o,d}},\;\;\;\;\;\forall \left(o,d,h_{o,d}\right)\in F(n,i,j)$$ (3)

where F(n,i,j) is users’ defined mathematical function to identify path $h_{o,d}$ from origin *o* to destination *d* turning from arm *i* to arm *j* at intersection *n*.

#### Users’ OD demand flow inputs

Users’ given OD demand flows can be represented by a set of binary variables and governed by Eq (4).

$$M^{2}T_{o,d}\geq M\beta_{o,d}\geq T_{o,d},\;\;\;\;\;\;\forall o\in \left\{1,2,\ldots ,O\right\},d\in \left\{1,2,\ldots ,D\right\}$$ (4)

where $\beta_{o,d}$ is a binary variable that represents OD flow, $T_{o,d}$. $\beta_{o,d}$ = “1” if the given $T_{o,d}$ > 0 (it is assumed that the minimum of $T_{o,d}$ in the model is 1.0 pcu/hr or should be zero otherwise) and *M* is an arbitrary large number (that should be greater than the largest given $T_{o,d}$ numerically).

#### Existence of path flows

If $T_{o,d}$ exists (> 0), a binary variable $\beta_{o,d}$ is “1” as required by Eq (4). When $\beta_{o,d}=1$, at least one path must exist to serve the respective demand flow in Eq (5). Multiple paths, which can also be modeled, share OD flows. In Eq (5), multiple paths exist when $\sum_{h_{o,d}=1}^{{\bar{H}}_{o,d}}\alpha_{o,d,h_{o,d}}>1$.

$$\sum_{h_{o,d}=1}^{{\bar{H}}_{a,b}}\alpha_{o,d,h_{o,d}}\geq \beta_{o,d},\;\;\;\;\;\;\;\forall o\in \left\{1,2,\ldots ,O\right\},d\in \left\{1,2,\ldots ,D\right\}$$ (5)

If the demand flow is given as zero, then $\beta_{o,d}$ is “0” and $\alpha_{o,d,h_{o,d}}$ = 0 by Eq (6). No path is required.

$$\beta_{o,d}\geq \alpha_{o,d,h_{o,d}},\;\;\;\;\;\forall o\in \left\{1,2,\ldots ,O\right\},d\in \left\{1,2,\ldots ,D\right\},h_{o,d}\in \left\{1,2,\ldots ,{\bar{H}}_{o,d}\right\}$$ (6)

#### Required lane markings for a path to serve OD pairs

In the present formulation, lane markings are optimized to connect network links and ensure that the required paths in Eq (7) serve a network’s OD flows.

$$\sum_{k=1}^{L_{n,i}}\delta_{n,i,j,k}\geq \alpha_{o,d,h_{o,d},}$$

$$\forall (n,i,j)\in F^{\prime }\left(o,d,h_{o,d}\right),o\in \left\{1,2,\ldots ,O\right\},d\in \left\{1,2,\ldots ,D\right\},h_{o,d}\in \left\{1,2,\ldots ,{\bar{H}}_{o,d}\right\}$$ (7)

where $\delta_{n,i,j,k}$ is a binary variable representing the lane marking for the turn from arm *i* to arm *j* on lane *k* at intersection *n* and $\alpha_{o,d,h_{o,d}}$ is the binary variable specifying the existence of path $h_{o,d}$ connecting OD pair *o* and *d*. When $\alpha_{o,d,h_{o,d}}$ = 1, corresponding lane markings for turns at intersections should be established on one of the approach lanes, *k*. Available paths can then be established. $F^{\prime }(o,d,h_{o,d})$ is a user defined mathematical function that identifies movement turns from arm i to arm j at intersection n along path $h_{o,d}$ connecting OD pair *o* and *d*.

#### Eliminating redundant lane markings

$$\Delta_{n,i,j,o,d,h_{o,d}}=\alpha_{o,d,h_{o,d},}$$

$$\forall \left(n,i,j\right)=F'\left(o,d,h_{o,d}\right),o\in \left\{1,2,\ldots ,O\right\},d\in \left\{1,2,\ldots ,D\right\},h_{o,d}\in \left\{1,2,\ldots ,{\bar{H}}_{o,d}\right\}$$ (8)

where $\alpha_{o,d,h_{o,d}}$ is a binary variable denoting the existence of path $h_{o,d}$. $\Delta_{n,i,j,o,d,h_{o,d}}$is an auxiliary binary variable representing a turn from arm *i* to arm *j* at intersection *n* establishing path $h_{o,d}$ for OD pair *o* and *d*. In Fig 1, from OD pair *o* and *d* using path $h_{o,d}$ at intersection *n* = 1, flows move from arm *i* = 1 to arm *j* = 3 and thus $\Delta_{n=1,i=1,j=3,o,d,h_{o,d}}$ = 1. Similar relationships can be established in Eq (8) for other intersections and paths. At intersection *n* = 24, there is no path flow turning left from arm *i* = 4 to arm *j* = 1 and thus $\Delta_{n=24,i=4,j=1,o,d,h_{o,d}}$ = 0. With auxiliary variable $\Delta_{n,i,j,o,d,h_{o,d}}$, we may then identify whether a specific lane marking $\delta_{n,i,j,k}$ for turns from arm *i* to arm *j* at intersection *n* is required. Eq (9) is required in the design framework for optimizing lane markings.

$$M^{2}\sum_{k=1}^{L_{n,i}}\delta_{n,i,j,k}\geq M\sum_{o=1}^{O}\sum_{d=1h}^{D}\sum_{h_{o,d}=1}^{{\bar{H}}_{o,d}}\Delta_{n,i,j,o,d,h_{o,d}}\geq \sum_{k=1}^{L_{n,i}}\delta_{n,i,j,k},$$

$$\forall n\in \{1,2,\ldots ,N\},i\in \{1,2,\ldots ,I_{n}\},j\in \{1,2,\ldots ,I_{n}\}$$ (9)

where *N* is the total number of intersections and $I_{n}$ is the total number of arms at intersection *n*.

#### Classifying the sequences and patterns of green duration times by transforming green signal phases for bandwidth evaluations

**Fig 2:**
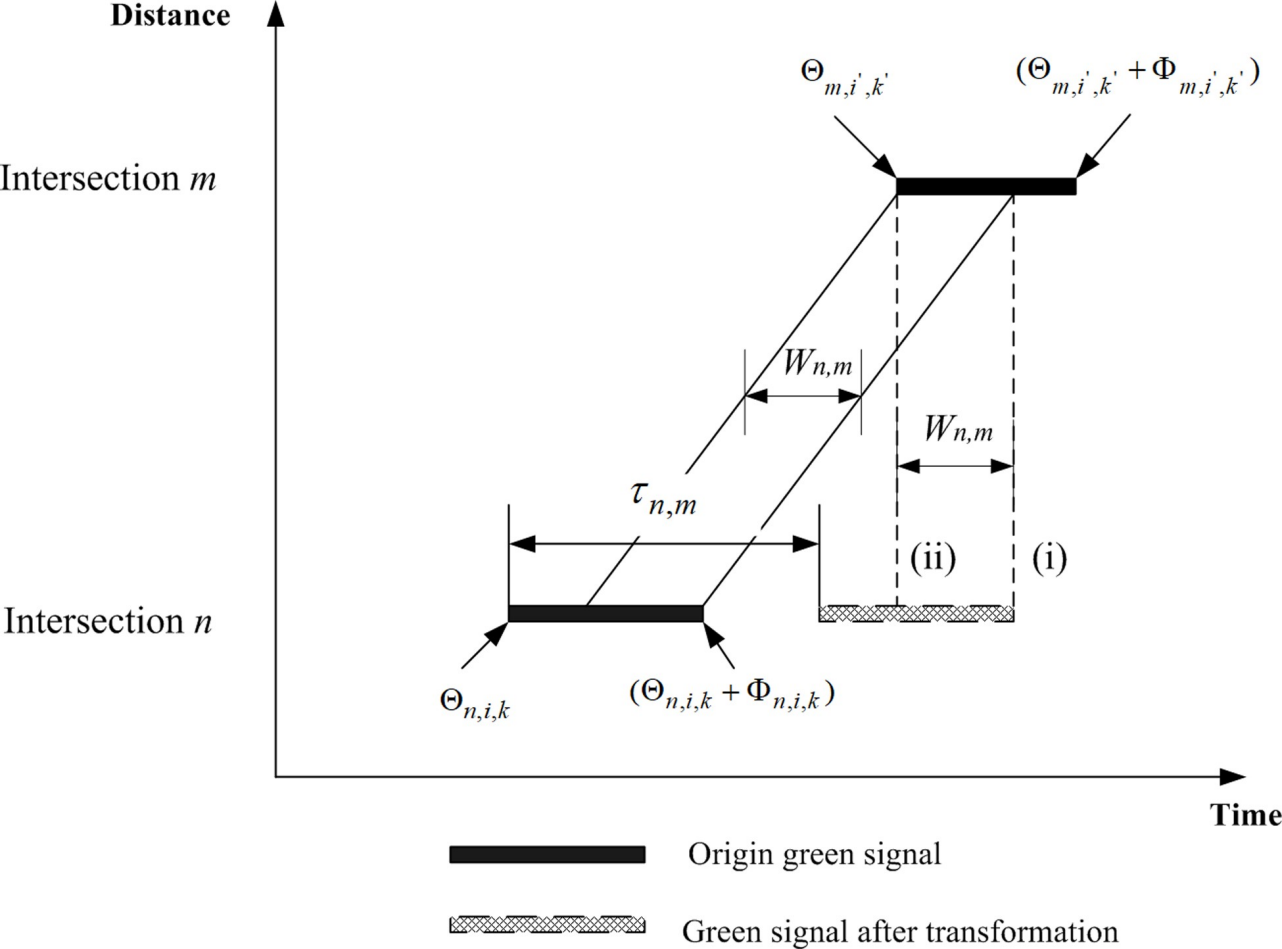
Typical green band between two intersections in a time-space diagram and transformation of a green signal phase for evaluating green bandwidth.

The shapes typical of the green bands between two consecutive intersections are illustrated in the time-space diagram given in Fig 2. To evaluate the green bandwidth in the proposed formulation, a linear transformation is performed to shift green phases from the upstream intersections horizontally along the time axis with the duration of average travel times from the upstream to downstream intersections. After the transformation, linear constraint sets can be developed based on simple rules to compare the start of green and end of green times for the upstream and downstream intersections. Auxiliary binary variables are introduced to monitor different outcomes of the comparisons for measuring the actual green bandwidths across pairs of consecutive upstream and downstream intersections.

As Fig 2 shows, there are two green signal phases for upstream intersection *n* and downstream intersection *m*, respectively. To evaluate the green bandwidth between the two green signal phases (i.e. thickened solid lines), a linear transformation is applied to shift the original green signal phase at upstream intersection *n* horizontally along the time axis with the amount of $\tau_{n,m}=S_{n,m}/{\bar{V}}_{n,m}$, which is the average travel time for vehicles leaving from upstream intersection *n* to reach downstream intersection *m*. $S_{n,m}$ is the distance separation between upstream *n* and downstream *m* and ${\bar{V}}_{n,m}$ is the average speed of vehicles moving from upstream *n* to downstream *m*. After performing the transformation, the revised green start time is $\Theta_{n,i,k}+\tau_{n,m}$. Similarly, the end of green time also moves to $\Theta_{n,i,k}+\Phi_{n,i,k}+\tau_{n,m}$. Two vertical dashed lines [(i) and (ii)] may be drawn: (i) from the end of green at upstream intersection *n* and (ii) from the start of green at downstream intersection *m*. Furthermore, bandwidth $W_{n,m}$ can be measured, representing the overlapping length of the two green signal phases [i.e. between the two vertical dashed lines (i) and (ii)] after the transformation. The practical transformation process is operated as follows.

The transformation process enables the evaluations of bandwidth found in the proposed programming approach. In the optimization process, traffic signal settings and green bandwidths can be varied and optimized with different and overlapping patterns used to generate the actual bandwidth *W*_*n*,*m*_. Linear constraint sets are developed to differentiate all of these cases and evaluate the corresponding bandwidth *W*_*n*,*m*_. In modeling, four conditions (I-IV) in the signal settings are considered. By comparing the transformed start of green time of upstream intersection *n*, which is $\Theta_{n,i,k}+\tau_{n,m}$, and the start of green time of downstream intersection *m*, $\Theta_{m,i',k'}+x_{n,m}$ (where *x*_*n*,*m*_ is an integer variable = 0, 1, 2, …), we may subtract the two green start times in Eq (10) to establish Condition I.

$$M\Lambda_{1,n,i,k,m,i^{\prime },k^{\prime }}\geq \Theta_{m,i^{\prime },k^{\prime }}+x_{n,m}-\Theta_{n,i,k}-\tau_{n,m}\geq M(\Lambda_{1,n,i,k,m,i^{\prime },k^{\prime }}-1),$$

$$\forall (m,i^{\prime })\in D(n,i,j),n\in \{1,2,\ldots ,N\},i\neq j\in \{1,2,\ldots ,I_{n}\},k\in \{1,2,\ldots ,L_{n,i}\},k^{\prime }\in \{1,2,\ldots ,L_{m,i^{\prime }}\}$$ (10)

where $\Lambda_{1,n,i,k,m,i',k'}$ is a binary variable (= 1 if the transformed start of green time for the vehicle movement of the upstream intersection (n,i,k) is earlier than the start of green time for the vehicle movement of the downstream intersection (m,i',k'), or = 0 if not). $L_{n,i}$ is the total number of approach lanes on arm *i* at intersection *n*. $L_{m,i^{\prime }}$ is the total number of approach lanes on arm $i^{\prime }$ at intersection *m*. A user’s defined mathematical function D(n,i,j) is to identify arm *i’* at downstream intersection *m*, (*m*, *i’*), taking the traffic from arm *i* to arm *j* at upstream intersection *n*.

To establish Condition II, a similar comparison as in Eq (10) is required for the two ends of the green times of upstream intersection *n* and downstream intersection *m*. In Eq (11), $\Lambda_{2,n,i,k,m,i',k'}$ is another binary variable to state the comparison result (= 1 if the transformed end of green time for the vehicle movement of the upstream intersection (n,i,k) is earlier than the end of green time for the vehicle movement of the downstream intersection (m,i',k'), or = 0 if not).

$$\begin{array}{l} M\Lambda_{2,n,i,k,m,i^{\prime },k^{\prime }}\geq \left(\Theta_{m,i^{\prime },k^{\prime }}+\Phi_{m,i^{\prime },k^{\prime }}\right)+x_{n,m}-\left(\Theta_{n,i,k}+\Phi_{n,i,k}\right)-\tau_{n,m}\geq M(\Lambda_{2,n,i,k,m,i^{\prime },k^{\prime }}-1),\\ \forall (m,i^{\prime })\in D(n,i,j),n\in \{1,2,\ldots ,N\},i\neq j\in \{1,2,\ldots ,I_{n}\},k\in \{1,2,\ldots ,L_{n,i}\},k^{\prime }\in \{1,2,\ldots ,L_{m,i^{\prime }}\} \end{array}$$ (11)

To establish Condition III, the comparison continues for the transformed end of green time for the vehicle movement of the upstream intersection (n,i,k) and the start of green time for the vehicle movement of the downstream intersection (m,i',k') in Eq (12). Again, binary variable $\Lambda_{3,n,i,k,m,i',k'}$ states whether the comparison result (= 1 if the start of green time for vehicle movement of the downstream intersection (m,i',k') is earlier than the transformed end of green time for the vehicle movement of the upstream intersection (n,i,k), or = 0 if not).

$$\begin{array}{l} M\Lambda_{3,n,i,k,m,i^{\prime },k^{\prime }}\geq \Theta_{n,i,k}+\Phi_{n,i,k}+\tau_{n,m}-\Theta_{m,i^{\prime },k^{\prime }}-x_{n,m}\geq M(\Lambda_{3,n,i,k,m,i^{\prime },k^{\prime }}-1),\\ \forall (m,i^{\prime })\in D(n,i,j),n\in \{1,2,\ldots ,N\},i\neq j\in \{1,2,\ldots ,I_{n}\},k\in \{1,2,\ldots ,L_{n,i}\},k^{\prime }\in \{1,2,\ldots ,L_{m,i^{\prime }}\} \end{array}$$ (12)

To establish Condition IV, a similar comparison is given to the transformed start of green time for the vehicle movement of the upstream intersection (n,i,k) and end of green time for the vehicle movement of the downstream intersection (m,i',k') in Eq (13).

$$\begin{array}{l} M\Lambda_{4,n,i,k,m,i^{\prime },k^{\prime }}\geq \Theta_{m,i^{\prime },k^{\prime }}+\Phi_{m,i^{\prime },k^{\prime }}+x_{n,m}-\Theta_{n,i,k}-\tau_{n,m}\geq M(\Lambda_{4,n,i,k,m,i^{\prime },k^{\prime }}-1),\\ \forall (m,i^{\prime })\in D(n,i,j),n\in \{1,2,\ldots ,N\},i\neq j\in \{1,2,\ldots ,I_{n}\},k\in \{1,2,\ldots ,L_{n,i}\},k^{\prime }\in \{1,2,\ldots ,L_{m,i^{\prime }}\} \end{array}$$ (13)

Another binary variable, $\Lambda_{4,n,i,k,m,i',k'}$, specifies whether the comparison result (= 1 if the transformed start of green time for the vehicle movement of the upstream intersection (n,i,k) is earlier than the end of green time for the vehicle movement of downstream intersection (m,i',k'), or = 0 if not).

#### Evaluating bandwidths for different patterns of signal timings

For the four different conditions identified in the previous section, six combinations in the (optimized) traffic signal settings are expected. To evaluate the bandwidths numerically, the six different combinations of the four conditions are considered with respect to the outcomes of the binary variables $\Lambda_{1,n,i,k,m,i',k'}$, $\Lambda_{2,n,i,k,m,i',k'}$, $\Lambda_{3,n,i,k,m,i',k'}$, and $\Lambda_{4,n,i,k,m,i',k'}$.

Case 1: Satisfying all Conditions (I-IV)

$$\begin{array}{l} M(4-\Lambda_{1,n,i,k,m,i^{\prime },k^{\prime }}-\Lambda_{2,n,i,k,m,i^{\prime },k^{\prime }}-\Lambda_{3,n,i,k,m,i^{\prime },k^{\prime }}-\Lambda_{4,n,i,k,m,i^{\prime },k^{\prime }})\geq W_{n,m}-\text{(}\Theta_{n,i,k}+\Phi_{n,i,k}+\\ \tau_{n,m}-\Theta_{m,i^{\prime },k^{\prime }}-x_{n,m})\geq -M(4-\Lambda_{1,n,i,k,m,i^{\prime },k^{\prime }}-\Lambda_{2,n,i,k,m,i^{\prime },k^{\prime }}-\Lambda_{3,n,i,k,m,i^{\prime },k^{\prime }}-\Lambda_{4,n,i,k,m,i^{\prime },k^{\prime }}),\\ \forall (m,i^{\prime })\in D(n,i,j),n\in \{1,2,\ldots ,N\},i\neq j\in \{1,2,\ldots ,I_{n}\},k\in \{1,2,\ldots ,L_{n,i}\},k^{\prime }\in \{1,2,\ldots ,L_{m,i^{\prime }}\} \end{array}$$ (14)

Case 2: Satisfying Conditions II, III, and IV

$$\begin{array}{l} M(3+\Lambda_{1,n,i,k,m,i^{\prime },k^{\prime }}-\Lambda_{2,n,i,k,m,i^{\prime },k^{\prime }}-\Lambda_{3,n,i,k,m,i^{\prime },k^{\prime }}-\Lambda_{4,n,i,k,m,i^{\prime },k^{\prime }})\geq W_{n,m}-\Phi_{n,i,k}\\ \geq -M(3+\Lambda_{1,n,i,k,m,i^{\prime },k^{\prime }}-\Lambda_{2,n,i,k,m,i^{\prime },k^{\prime }}-\Lambda_{3,n,i,k,m,i^{\prime },k^{\prime }}-\Lambda_{4,n,i,k,m,i^{\prime },k^{\prime }}), \end{array}$$

$$\forall (m,i^{\prime })\in D(n,i,j),n\in \{1,2,\ldots ,N\},i\neq j\in \{1,2,\ldots ,I_{n}\},k\in \{1,2,\ldots ,L_{n,i}\},k^{\prime }\in \{1,2,\ldots ,L_{m,i^{\prime }}\}$$ (15)

Case 3: Satisfying Conditions I, III, and IV

$$\begin{array}{l} M(3-\Lambda_{1,n,i,k,m,i^{\prime },k^{\prime }}+\Lambda_{2,n,i,k,m,i^{\prime },k^{\prime }}-\Lambda_{3,n,i,k,m,i^{\prime },k^{\prime }}-\Lambda_{4,n,i,k,m,i^{\prime },k^{\prime }})\geq W_{n,m}-\Phi_{m,i^{\prime },k^{\prime }}\\ \geq -M(3-\Lambda_{1,n,i,k,m,i^{\prime },k^{\prime }}+\Lambda_{2,n,i,k,m,i^{\prime },k^{\prime }}-\Lambda_{3,n,i,k,m,i^{\prime },k^{\prime }}-\Lambda_{4,n,i,k,m,i^{\prime },k^{\prime }}),\\ \forall (m,i^{\prime })\in D(n,i,j),n\in \{1,2,\ldots ,N\},i\neq j\in \{1,2,\ldots ,I_{n}\},k\in \{1,2,\ldots ,L_{n,i}\},k^{\prime }\in \{1,2,\ldots ,L_{m,i^{\prime }}\} \end{array}$$ (16)

Case 4: Satisfying Conditions I, II, and IV

$$\begin{array}{l} M(3-\Lambda_{1,n,i,k,m,i^{\prime },k^{\prime }}-\Lambda_{2,n,i,k,m,i^{\prime },k^{\prime }}+\Lambda_{3,n,i,k,m,i^{\prime },k^{\prime }}-\Lambda_{4,n,i,k,m,i^{\prime },k^{\prime }})\geq W_{n,m}\\ \geq -M(3-\Lambda_{1,n,i,k,m,i^{\prime },k^{\prime }}-\Lambda_{2,n,i,k,m,i^{\prime },k^{\prime }}+\Lambda_{3,n,i,k,m,i^{\prime },k^{\prime }}-\Lambda_{4,n,i,k,m,i^{\prime },k^{\prime }}), \end{array}$$

$$\forall (m,i^{\prime })\in D(n,i,j),n\in \{1,2,\ldots ,N\},i\neq j\in \{1,2,\ldots ,I_{n}\},k\in \{1,2,\ldots ,L_{n,i}\},k^{\prime }\in \{1,2,\ldots ,L_{m,i^{\prime }}\}$$ (17)

Case 5: Satisfying Conditions III and IV

$$\begin{array}{l} M(2+\Lambda_{1,n,i,k,m,i^{\prime },k^{\prime }}+\Lambda_{2,n,i,k,m,i^{\prime },k^{\prime }}-\Lambda_{3,n,i,k,m,i^{\prime },k^{\prime }}-\Lambda_{4,n,i,k,m,i^{\prime },k^{\prime }})\geq W_{n,m}\text{-(}\Theta_{m,i^{\prime },k^{\prime }}+\Phi_{m,i^{\prime },k^{\prime }}\\ +x_{n,m}-\Theta_{n,i,k}-\tau_{n,m})\geq -M(2+\Lambda_{1,n,i,k,m,i^{\prime },k^{\prime }}+\Lambda_{2,n,i,k,m,i^{\prime },k^{\prime }}-\Lambda_{3,n,i,k,m,i^{\prime },k^{\prime }}-\Lambda_{4,n,i,k,m,i^{\prime },k^{\prime }}),\\ \forall (m,i^{\prime })\in D(n,i,j),n\in \{1,2,\ldots ,N\},i\neq j\in \{1,2,\ldots ,I_{n}\},k\in \{1,2,\ldots ,L_{n,i}\},k^{\prime }\in \{1,2,\ldots ,L_{m,i^{\prime }}\} \end{array}$$ (18)

Case 6: Satisfying Condition III only

$$\begin{array}{l} M(1+\Lambda_{1,n,i,k,m,i^{\prime },k^{\prime }}+\Lambda_{2,n,i,k,m,i^{\prime },k^{\prime }}-\Lambda_{3,n,i,k,m,i^{\prime },k^{\prime }}+\Lambda_{4,n,i,k,m,i^{\prime },k^{\prime }})\geq W_{n,m}\\ \geq -M(1+\Lambda_{1,n,i,k,m,i^{\prime },k^{\prime }}+\Lambda_{2,n,i,k,m,i^{\prime },k^{\prime }}-\Lambda_{3,n,i,k,m,i^{\prime },k^{\prime }}+\Lambda_{4,n,i,k,m,i^{\prime },k^{\prime }}), \end{array}$$

$$\forall (m,i^{\prime })\in D(n,i,j),n\in \{1,2,\ldots ,N\},i\neq j\in \{1,2,\ldots ,I_{n}\},k\in \{1,2,\ldots ,L_{n,i}\},k^{\prime }\in \{1,2,\ldots ,L_{m,i^{\prime }}\}$$ (19)

**Fig 3:**
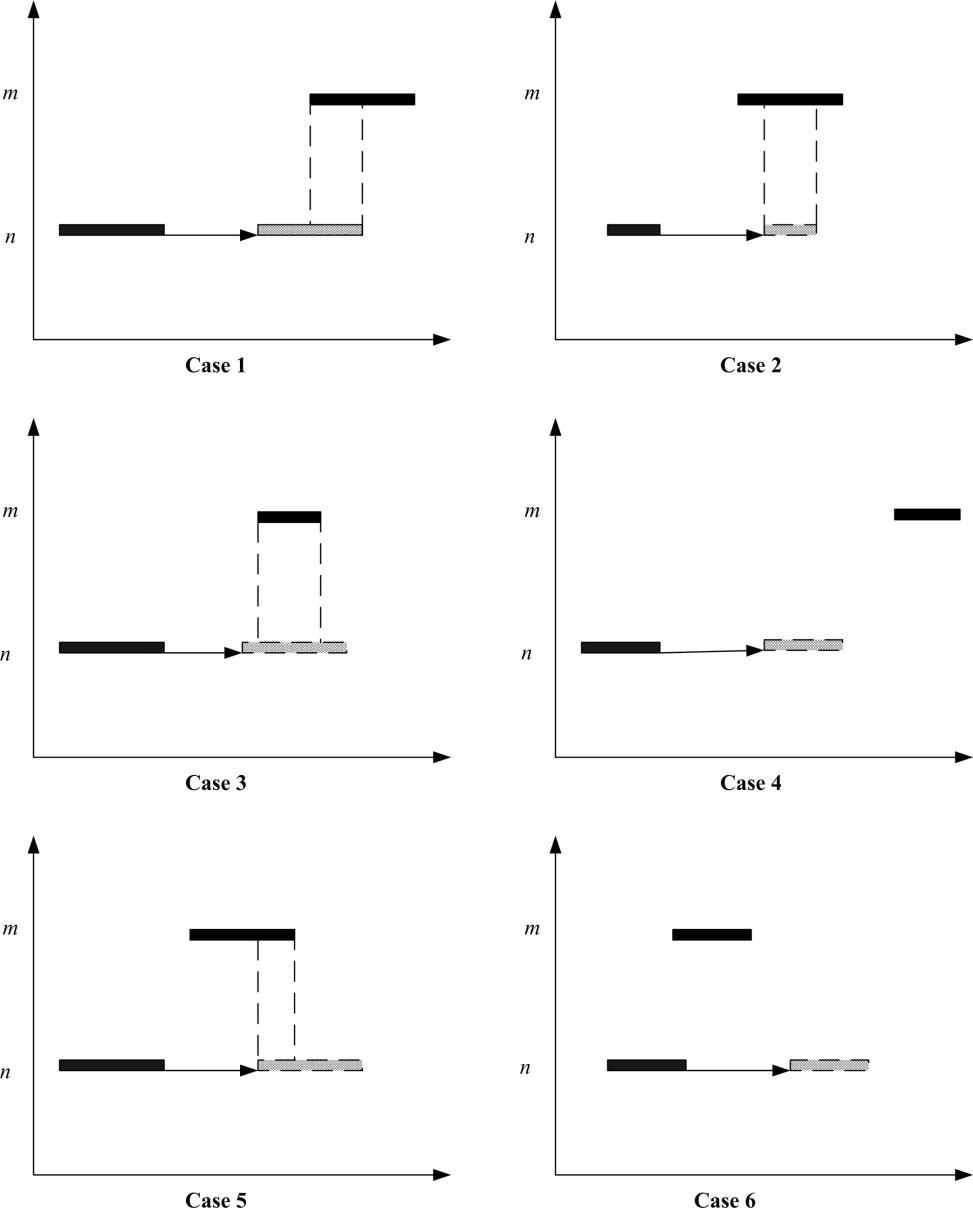
Six different patterns of traffic signal settings after transformation.

Cases 1–6 given in Fig 3 are the different combinations in the patterns for the starts and ends of green signal phases at upstream intersection *n* and downstream intersection *m*. Cases 1, 2, 3, and 5 are likely to generate effective green bandwidths (i.e. green signal phases can overlap), while Cases 4 and 6 do not generate effective bandwidths (no overlap) in the solution process.

#### Maximum length of green bandwidth

Apart from mathematical constraint sets developed in previous sections, which relate the adjacent upstream and downstream intersections, it must be noted that the actual (green) bandwidth should always be shorter than or equal to the green duration times operating at individual intersections for the traffic moving along the selected major routes and OD pairs. Eqs (20) and (21) are given to restrict the maximum (green) bandwidth in the solution process.

$$\text{0}\leq W_{n,m}\leq \Phi_{n,i,k},\;\;\;\;\;\forall (m,i^{\prime })\in D(n,i,j),n\in \{1,2,\ldots ,N\},i\neq j\in \{1,2,\ldots ,I_{n}\},k\in \{1,2,\ldots ,L_{n,i}\}$$ (20)

$$\text{0}\leq W_{n,m}\leq \Phi_{m,i^{\prime },k^{\prime }},\;\;\forall (m,i^{\prime })\in D(n,i,j),n\in \{1,2,\ldots ,N\},i\neq j\in \{1,2,\ldots ,I_{n}\},k^{\prime }\in \{1,2,\ldots ,L_{m,i^{\prime }}\}$$ (21)

#### Bandwidths for coordinating upstream and downstream intersections

To put forward an effective coordination between upstream and downstream traffic signal settings, maximizing the green bandwidth is set as the objective in the present formulation. Before bandwidth can be optimized in the objective function, we must ensure that the bandwidth exists in which all transformed upstream green signal phases can overlap the downstream green signal phases. There are two possibilities, governed by Eqs (22) and (23).

If the transformed start of green time for the vehicle movement of the upstream intersection (n,i,k) is earlier than the start of green time for the vehicle movement of the downstream intersection (m,i',k'), then to ensure that the two signal phases can overlap to produce effective green bandwidth, as in Cases 1 and 3, we need to force the transformed end of green time at the upstream intersection (n,i,k) to be later than the start of green time at the downstream intersection (m,i',k'). The required constraint set is given in Eq (22).

$$\begin{array}{l} 1-\Lambda_{1,n,i,k,m,i^{\prime },k^{\prime }}\geq \Lambda_{3,n,i,k,m,i^{\prime },k^{\prime }}-1\geq \Lambda_{1,n,i,k,m,i^{\prime },k^{\prime }}-1\text{,}\\ \forall (m,i^{\prime })\in D(n,i,j),n\in \{1,2,\ldots ,N\},i\neq j\in \{1,2,\ldots ,I_{n}\},k\in \{1,2,\ldots ,L_{n,i}\},k^{\prime }\in \{1,2,\ldots ,L_{m,i^{\prime }}\} \end{array}$$ (22)

If the transformed start of green time at the upstream intersection (n,i,k) is later than the start of green time at the downstream intersection (m,i',k'), as in Cases 2 and 5, then the end of green time at the downstream intersection (m,i',k') must be later than the transformed start of green at the upstream intersection (n,i,k). The related constraint set is given in Eq (23).

$$\Lambda_{1,n,i,k,m,i^{\prime },k^{\prime }}\geq \Lambda_{4,n.i,k,m,i^{\prime },k^{\prime }}-1\geq -\Lambda_{1,n,i,k,m,i^{\prime },k^{\prime }},$$

$$\forall (m,i^{\prime })\in D(n,i,j),n\in \{1,2,\ldots ,N\},i\neq j\in \{1,2,\ldots ,I_{n}\},k\in \{1,2,\ldots ,L_{n,i}\},k^{\prime }\in \{1,2,\ldots ,L_{m,i^{\prime }}\}$$ (23)

**Fig 4:**
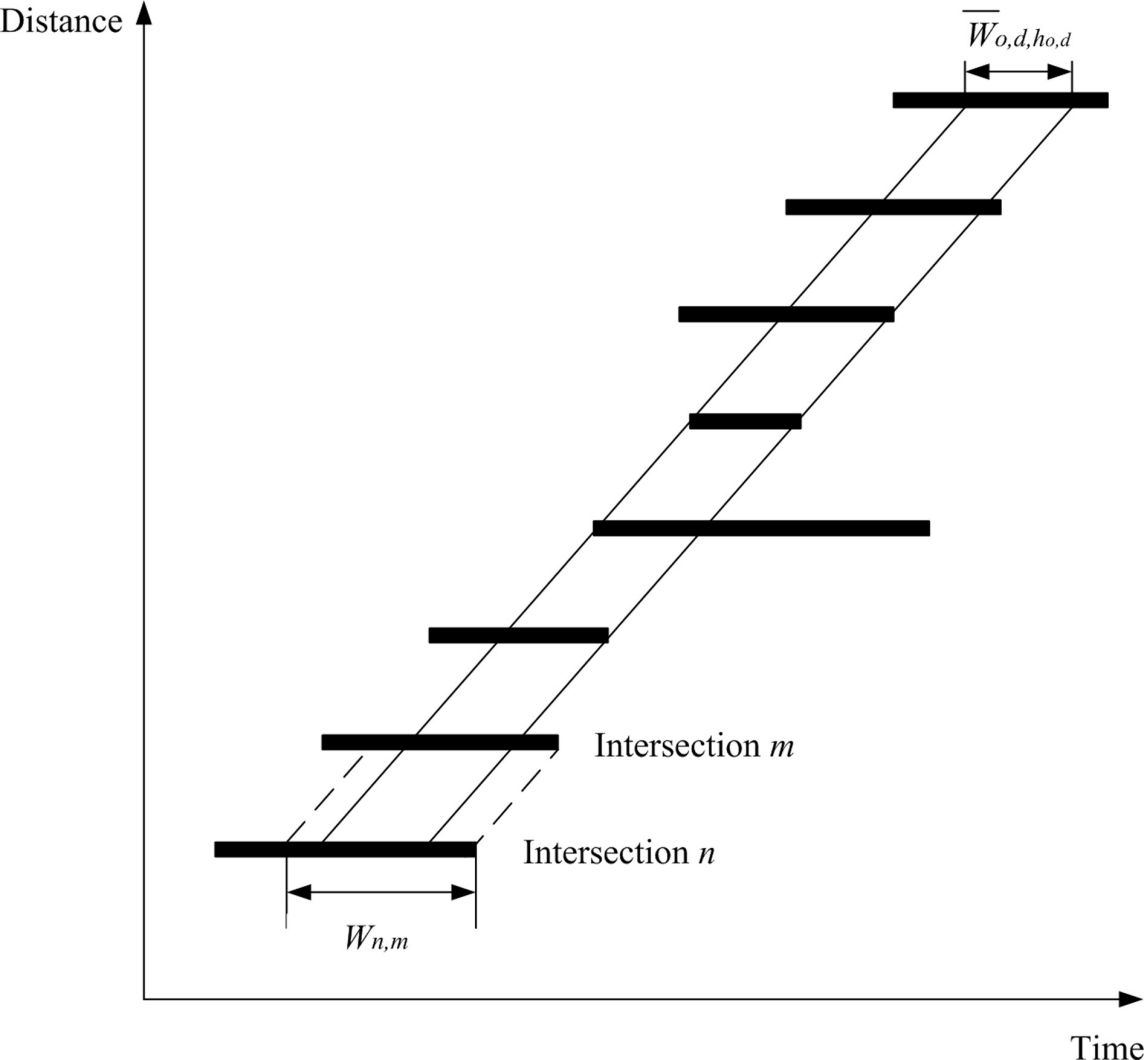
Overall bandwidth ${\bar{W}}_{o,d,h_{o,d}}$for a selected path $h_{o,d}$.

In this study, maximum bandwidths are designed to be the maximum length that can be optimized “in common” from the overlapping bandwidths shown in Fig 4. The overall common bandwidth is represented by ${\bar{W}}_{o,d,h_{o,d}}$ along the selected path $h_{o,d}$ from the selected origin *o* to the selected destination *d*. The constraint set in Eq (24) is required to obtain the actual common bandwidth from all pairs of upstream and downstream intersections.

$${\bar{W}}_{o,d,h_{o,d}}\leq W_{n,m}\text{,}$$

$$\forall (m,i^{\prime })\in D(n,i,j),(n,i,j)\in F^{\prime }(o,d,h_{o,d}),o\in \left\{1,\ldots ,O\right\},d\in \left\{1,\ldots ,D\right\},h_{o,d}\in \left\{1,\ldots ,{\bar{H}}_{o,d}\right\}$$ (24)

#### Total intersection delay by approximated delay function

A 3-term Webster’s delay formula was developed (but could be simplified and approximated by using the first two terms of the original formula) for the uniform and random delays at signalized intersections [50]. Webster’s delay formula grows infinitely and unrealistically large when the degree of saturation approaches 100%. Then, a proposal emerged for developing a coordinate transformation technique to predict the intersection delays that are subject to time-varying traffic demand and capacity for both under- and over-saturated conditions [51]. According to the TRANSYT traffic model [21, 22, 35, 52], link travel times include two components: link cruise time and end of link delay. The cruise time on a link has been considered to be the free flow travel time along the link. The delay at the end of link consists of uniform delay due to alternations of green and red traffic signals and random and oversaturation delay due to random traffic arrivals and degree of saturation > 100%, respectively [53]. Uniform delay can be calculated directly using the first term of Webster’s equation that depends on cycle length, the proportion of a signal cycle, which is effectively green, and the degree of saturation. Random and over-saturation delay can be evaluated based on a sheared delay formula [24], depending on link capacity, initial queue length, degree of saturation, analysis period, and the constant in the Pollaczek-Khintchine (P-K) formula [54]. The P-K constant in the sheared delay formula was calibrated for use in a coordinated network in Hong Kong [55].

Obviously, the shear delay formula calculating delays at the ends of links are non-linear functions. Adding those uniform delay and random and oversaturation delay (formula) directly to the proposed formulation may turn the entire design problem into a binary-mixed-integer-non-linear-program, which is very difficult to solve [26, 28]. The linear approximation technique and simple functional form were successfully applied to predict total delays in a signalized system [56]. In this study, a similar concept and linearization technique is applied to evaluate the delay time at the end of a lane that is related to its lane flow (including turning and straight-ahead movements), proportion of a signal cycle which is effectively green, and degree of saturation. For slowly varying non-linear delay function, it is possible to decouple the original product terms into individual linear terms for approximations. In the present formulation, total lane flow, ratio of lane flow to saturation flow, effective green duration time, and cycle length are extracted from the lane-based model to be the linearized function variables for predicting delays at ends of lanes. Eq (25) is the approximated function.

$$\varphi_{n,i,k}=\eta_{c}\zeta +\eta_{e}(\Phi_{n,i,k}+e\zeta )+\eta_{y}y_{n,i,k}+\eta_{q}\sum_{\begin{array}{l} j=1\\ j\neq i \end{array}}^{I_{n}}q_{n,i,j,k}\text{,}$$

$$\forall n\in \{1,2,\ldots ,N\},i\in \{1,2,\ldots ,I_{n}\},k\in \{1,2,\ldots ,L_{n,i}\}$$ (25)

where $\varphi_{n,i,k}$ is the total delay time at the end of lane *k* from arm *i* at intersection *n*. $\eta_{c}$, $\eta_{e}$, $\eta_{y}$, and $\eta_{q}$ are respectively the numerical coefficients for weighting cycle length, effective green duration time, ratio of lane flow-to-saturation flow, and lane flow to predict end of lane delays.

#### Lane travel time by cruise time and end of lane delay

**Fig 5:**
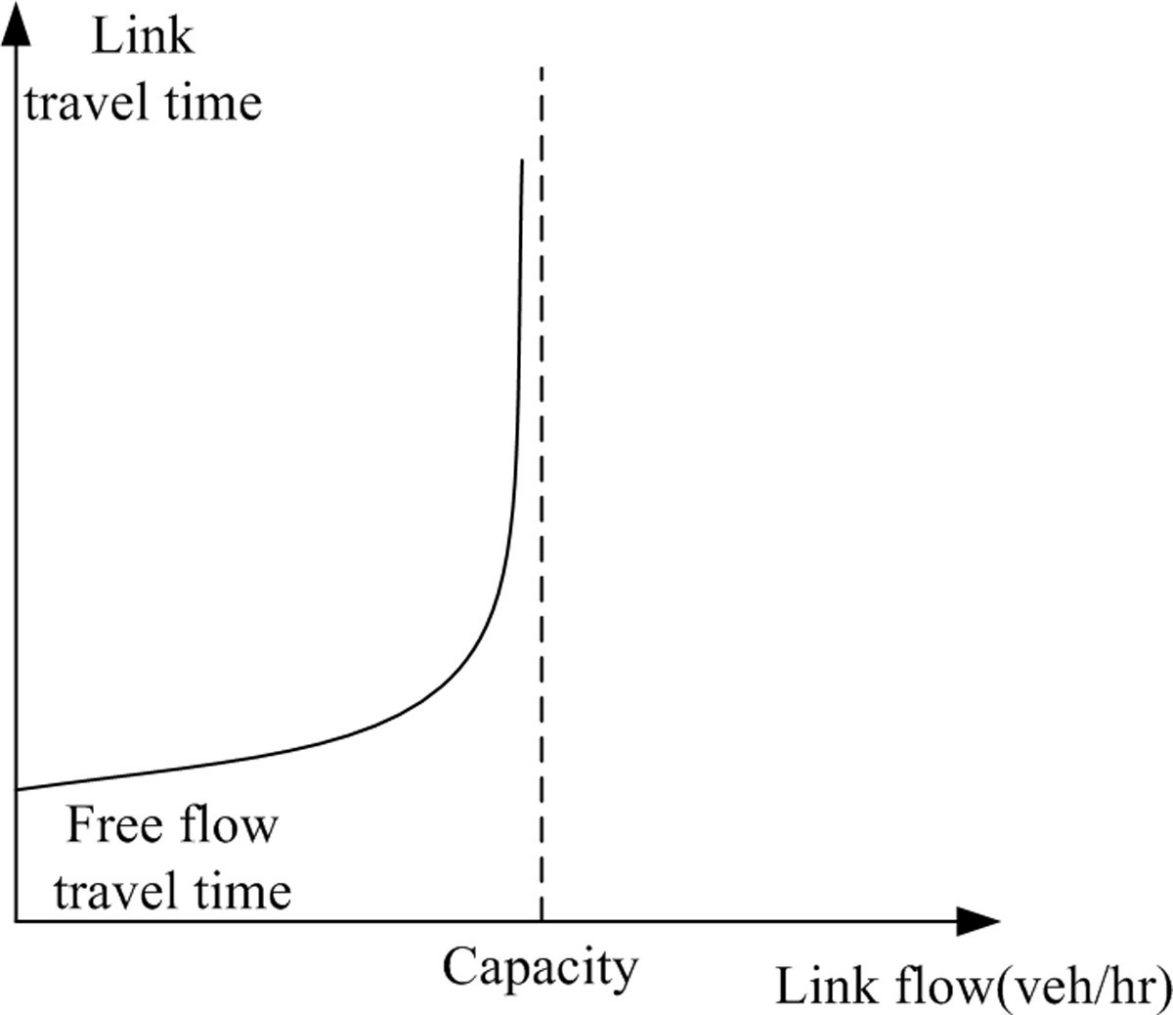
Typical link performance function (Source: Fig 1.8 [57]).

In the present formulation, lane marking patterns are optimized to establish network link connections. Consecutive intersection pairs are then linked to serve the given OD flows that could be distributed onto available paths. To satisfy the flow conservation requirements, OD flows and the sum of the associated (available) path flows should be equalized. In the solution process, path travel times should be evaluated while optimizing path flows onto different paths. Shorter paths in terms of path travel times through different lanes and intersections should be used while longer paths should not be chosen by users. According to Sheffi [57], a link performance function for a typical approach to a signalized intersection, as shown in Fig 5, captures both the time spent in traveling along the approach and the delay at the downstream intersection. In practice, a simplified Bureau of Public Roads function depending on flow-to-capacity ratio is commonly used. Obviously, such link performance function is non-linear in nature due to a power term applied to the flow-to-capacity ratio resulting with asymptotic link travel time values when link flows approach their capacity values (i.e. degree of saturation is near 100.0%). In the present linear formulation, directly applying the link performance function to calculate path travel times may turn the entire formulation into a non-linear problem that is difficult to solve.

In the proposed formulation, the lane travel time, $\gamma_{n,i,k}$, in Eq (26) is the sum of (1) vehicle traversing time on the used lane between upstream intersection *n* and downstream intersection *m*, $\tau_{n,m}=S_{n,m}/{\bar{V}}_{n,m}$ (i.e. the cruise time) and (2) total delay at the end of the lane, $\varphi_{n,i,k}$.

$$\gamma_{n,i,k}=\tau_{n,m}+\phi_{n,i,k},\;\;\forall (m,i^{\prime })\in D(n,i,j),n\in \{1,2,\ldots ,N\},i\neq j\in \{1,2,\ldots ,N\},k\in \{1,2,\ldots ,L_{n,i}\}$$ (26)

**Fig 6:**
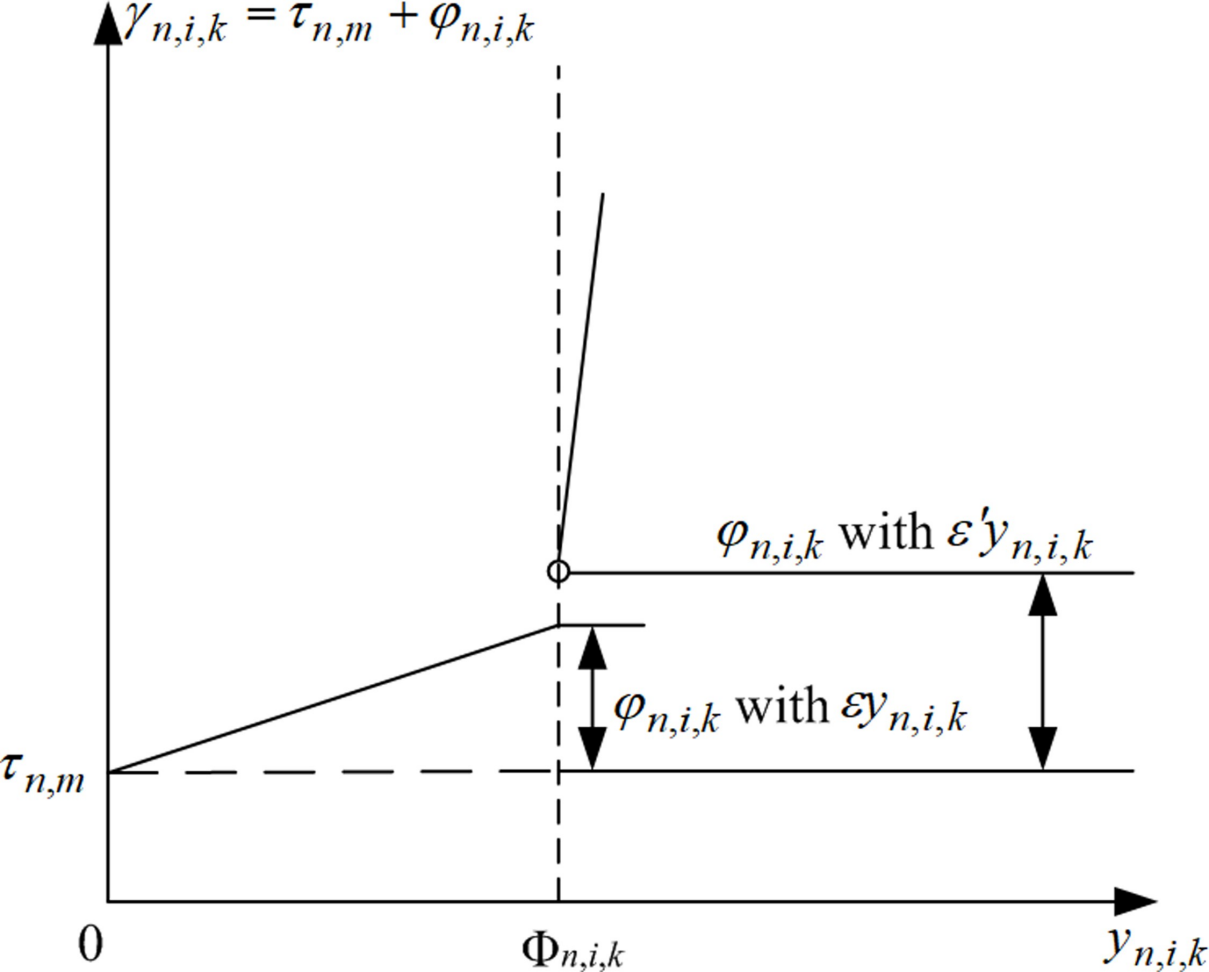
Proposed 2-piece linearized function for evaluating $\gamma_{n,i,k}$.

In this section, linear constraint sets are developed to evaluate the travel times along lanes using piecewise linear functions. A two-piece linearized function is proposed as a replacement to maintain the linearity in the formulation, as shown in Fig 6. To match the original shape of the non-linear link performance function in Fig 5, we have to ensure that a sharp increase in the travel time along a lane if the lane flow exceeds the lane capacity given in Fig 6. The threshold position is near the flow-to-capacity ratio = 100% (i.e. demand flow = capacity) as given by the original link performance function. In traffic signal controls, the degree of saturation is known as the flow-to-capacity ratio, which is equal to the lane flow factor multiplying cycle length and dividing by the effective green duration time. The corresponding threshold position appears when the flow factor equals the proportion of a signal cycle that is effectively green. In Fig 6, two linear lines, based on two different increasing slopes, simulate the original curve shape of the link performance function for evaluating lane travel time $\gamma_{n,i,k}$. In Eq (27), an auxiliary binary variable $\Pi_{n,i,k}$ for lane *k* from arm *i* at intersection *n* is forced to be zero when the flow factor *y*_*n*,*i*,*k*_ is smaller than or equal to the proportion of a signal cycle which is effectively green, $\Phi_{n,i,k}$ for unsaturation conditions. Or $\Pi_{n,i,k}=1$ when $y_{n,i,k}>\Phi_{n,i,k}$ for over-saturation conditions.

$$M\Pi_{n,i,k}\geq y_{n,i,k}-\Phi_{n,i,k}>M(\Pi_{n,i,k}-1),\;\forall n\in \{1,2,\ldots ,N\},i\in \{1,2,\ldots ,I_{n}\},k\in \{1,2,\ldots ,L_{n,i}\}$$ (27)

Based on the binary results of $\Pi_{n,i,k}$, Eqs (28) and (29) are developed to control the numerical coefficients used to weight the lane flow factor in the proposed linear delay function in Eq (25) to evaluate the total delays at the ends of lanes. Whenever $\Pi_{n,i,k}=0$, Eq (28) forces the numerical coefficient $\eta_{y}=\varepsilon$, which is the first segment of the 2-piece linearized function with a gentle increasing slope ε. Or $\Pi_{n,i,j}=1$, Eq (29) forces $\eta_{y}=\varepsilon^{\prime }$ which is the second segment of the 2-piece linearized function with a sharper increasing slope ε'.

$$M\Pi_{n,i,k}\geq \eta_{y}-\varepsilon \geq -M\Pi_{n,i,k},\;\;\forall n\in \{1,\ldots ,N\},i\in \{1,\ldots ,I_{n}\},k\in \{1,\ldots ,L_{n,i}\}$$ (28)

$$M\left(1-\Pi_{n,i,k}\right)\geq \eta_{y}-\varepsilon^{\prime }\geq -M\left(1-\Pi_{n,i,k}\right),\;\forall n\in \{1,2,\ldots ,N\},i\in \{1,2,\ldots ,I_{n}\},k\in \{1,2,\ldots ,L_{n,i}\}$$ (29)

where $\varepsilon <\varepsilon^{\prime }$ numerically.

#### Ensuring identical path travel times for used paths connecting origins and destinations

In a network, users tend to use the shortest path(s) to travel between OD pairs. In the present formulation, paths are composed by linking up series of intersections on different approach lanes based on lane marking patterns. To assign path flows onto different available paths, path travel times, and associated total delays at intersections should be considered. In Eq (30), path travel time $\chi_{o,d,h_{o,d}}$ connecting origin *o* and destination *d* along path $h_{o,d}$ equals the sum of all involved lane travel times $\gamma_{n,i,k}$ from Eqs (25)–(29) for different traffic conditions.

$$\chi_{o,d,h_{o,d}}=\sum_{\left(n,i,k)\in Κ(o,d,h_{o,d}\right)}\gamma_{n,i,k},\;\forall (n,i,k)\in Κ(o,d,h_{o,d}),o\in \{1,\ldots ,O\},d\in \{1,\ldots ,D\},h_{o,d}\in \{1,\ldots ,{\bar{H}}_{o,d}\}$$ (30)

where $\chi_{o,d,h_{o,d}}$ is path travel time from origin *o* to destination *d* along the path $h_{o,d}$. In the present optimization framework, multiple paths may connect the given OD pairs and share OD flows. To ensure identical path travel times along all used paths (i.e. path flows are non-zero) for a particular OD pair, Eq (31) is required to equalize the path travel times $\chi_{o,d,h_{o,d}}=\chi_{o,d,h{'}_{o,d}}$ whenever paths $h_{o,d}$ and ${h^{\prime }}_{o,d}$ exist simultaneously. In addition, the two required binary variables $\alpha_{o,d,h_{o,d}}$and $\alpha_{o,d,h{'}_{o,d}}$ are obtained from Eq (2). To ensure that all longer paths are unfavorable in route choices, Eq (31) can be used to guarantee that path travel times are longer when paths are not chosen by users. Path flows along those longer paths must be zero. With Eq (31) in the proposed formulation, the resultant traffic flow pattern satisfies the Wardropian user-equilibrium principle in which no user in the network can unilaterally change routes to reduce travel times [58].

$$M(\alpha_{o,d,{h^{\prime }}_{o,d}}-\text{1})\leq \chi_{o,d,h_{o,d}}-\chi_{o,d,{h^{\prime }}_{o,d}}\leq M(\text{1}-\alpha_{o,d,h_{o,d}})\text{,}$$

$$\forall o\in \left\{1,2,\ldots ,O\right\},d\in \left\{1,2,\ldots ,D\right\},h_{o,d}\in \left\{1,2,\ldots ,{\bar{H}}_{o,d}\right\},{h^{\prime }}_{o,d}\neq h_{o,d}\in \left\{1,2,\ldots ,{\bar{H}}_{o,d}\right\}$$ (31)

### Objective function for maximizing green bandwidths

Green bandwidth maximization enables the coordination of traffic signal settings in a network system. With such an approach, it is possible to determine the best offsets of green start times for all involved signal phases along a selected major path. In this study, bandwidth across all pairs of consecutive intersections along selected major paths can be evaluated linearly. Other linear constraint sets for configuring network link connections, described above in an effort to outline a solution region for linear optimization, are setting up individual signal-controlled intersections with lane marking designs and safe traffic signal timings [20, 22], evaluating total delays at ends of lanes, estimating lane travel times, and ensuring identical travel times for used paths. In the present formulation, lane markings are the key binary type variables to be optimized for the design of network configuration. To take full advantage of the design features of current lane-based methods, we need to maintain a linear platform in the optimization framework. Maximizing the green bandwidth is thus chosen as the objective for optimization in the proposed study. The problem can then be formulated as a BMILP problem encapsulating the lane markings, link connections for defining network configurations, traffic signal settings including offsets for coordinating signal timings across adjacent intersections, and green bandwidths for a signalized network design problem. A new approach is introduced to enable the design of network configurations, relaxing lane markings as design variables to support network link connections and link up adjacent intersections. As for the objective function, the overall common bandwidth is applied for establishing the linear objective function in Eq (32) for optimization.

By maximizing the green bandwidths, it is possible to obtain optimized signal timings, lane marking patterns, lane flows, and path flows. To solve the proposed optimization problem, users aiming to receive green bands should provide their origin nodes, destination nodes, and selected major paths with their preferences. The selected major paths become more attractive for users when connecting their OD pairs. In a study network, OD demand flows are given as users’ input data. The resultant lane markings on approach lanes at various intersections should be compiled to configure the network’s link connections. Assigned lane flows that are compatible with the optimized set of lane markings can be optimized to match their respective path flows. In addition, all path flows can be combined to equalize the given OD demand flow patterns, thus satisfying the requirements for flow conservation. Optimized signal timings are effective and efficient for practical operations. Better linkages between pairs of upstream and downstream intersections can be established by providing proper offsets. Mathematically, the bandwidth maximization problem can be formulated as a BMILP:

$$\underset{selected(o,d,h_{od})}{\text{Maximize}}\;\;\;({\bar{W}}_{o,d,h_{o,d}}+z_{o,d,h_{o,d}}{\bar{W}}_{d,o,h_{d,o}})$$ (32)

subject to linear constraints in (1–31) and the 13 standard sets of lane-based constraints for individual intersections given in [25, 27]. Also, $z_{o,d,h_{o,d}}$ in Eq (32) is the set of users’ given numerical weightings ($=\frac{{\bar{W}}_{d,o{,}_{h_{d,o}}}}{{\bar{W}}_{o,d,h_{o,d}}}$) for two opposite inbound and outbound green bandwidths in the optimization process according to their relative importance. This BMILP problem can be solved by a standard branch-and-bound routine. A numerical example with four signalized intersections and four OD pairs is used to demonstrate the proposed design method in next section. Lane markings and signal timings can be optimized in a combined manner to generate both an efficient network and effective traffic signal settings. Assigned lane flows are optimized to satisfy the requirements for flow conservation.

## Optimization results from a case study network with four intersections

**Fig 7:**
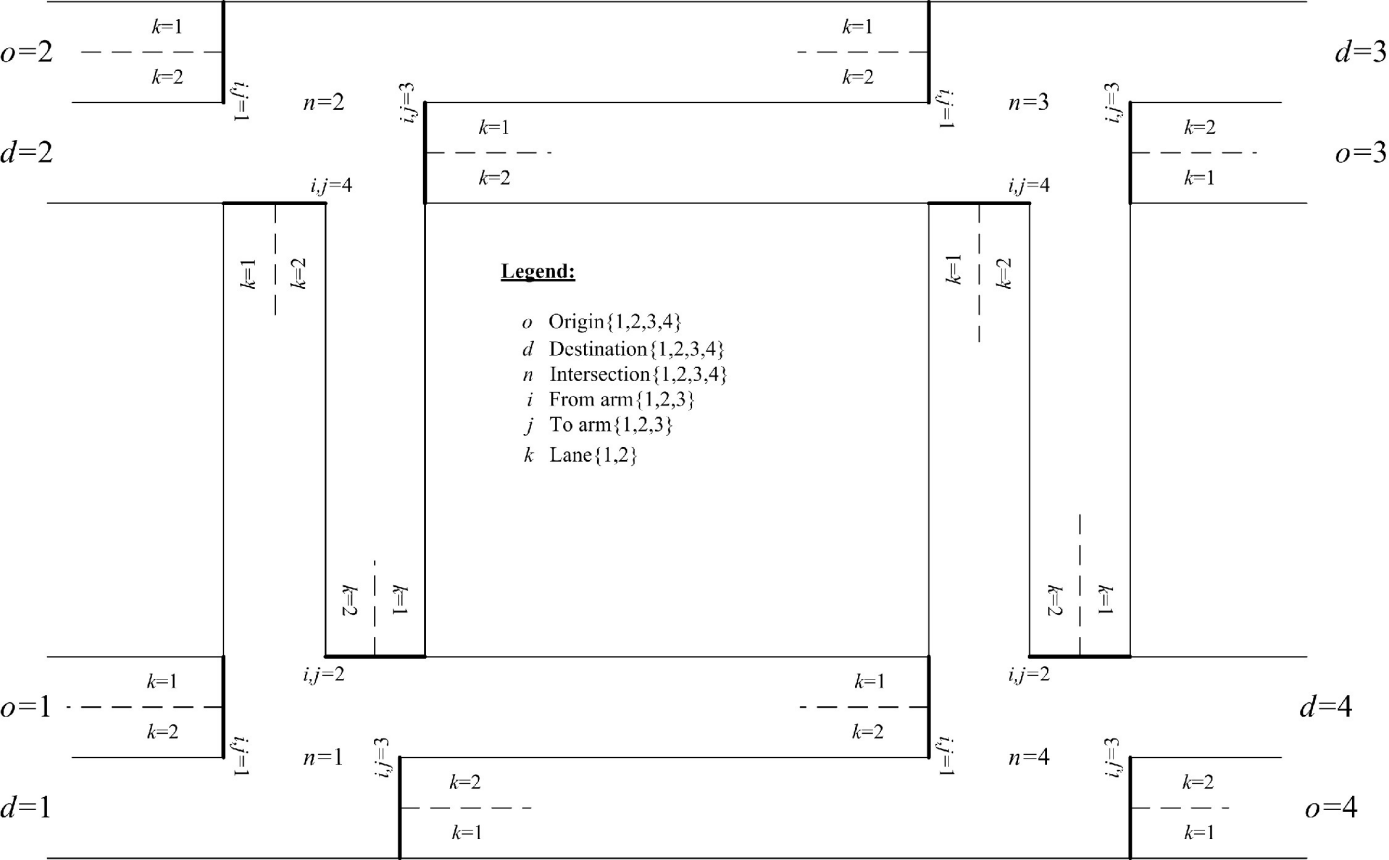
Settings of the case study signal-controlled network with four signalized intersections.

In this section, a four-intersection signal-controlled network is modeled with *N* = 4. Lane markings for all intersections are to be optimized to configure network links and establish link connectivity. Fig 7 presents the shape of the study network. The established lane markings are optimized for the link connections and the network configuration. There are two approach lanes, *L*_*n*,*i*_ = 2, for all arms *I* = *J* = 4 at all intersections, *n* = 1, 2, 3, and 4. All left-hand lanes *k* = 1 are nearside lanes and right-hand lanes *k* = 2 are non-nearside lanes with saturation flows of ${\bar{S}}_{n,i,k=1}$=1,965 pcu/h and ${\bar{S}}_{n,i,k=2}$=2,105 pcu/h for straight-ahead movements, respectively. Lane marking arrows, assigned lane flows, and signal timings are free to be optimized for all approach lanes at the intersections. Saturation flows for straight-ahead movement are revised to account for their lane turning proportions, which are based on optimized lane flows. There are four nodes serving as origins (*o* = 1, 2, 3, and 4) and also as destinations (*d* = 1, 2, 3, and 4). Numerical OD flows, $T_{o,d}$, are users’ inputs given in Table 1. In this case study, all the OD flows may have two different feasible paths connecting every OD pair if the necessary lane markings are designed. For example, from origin *o* = 1 to destination *d* = 3, the input demand flow is $h_{o=1,d=3}=500$ pcu/h that could be assigned to use path 1 ($h_{o=1,d=3}=1$) through intersections *n* = 1, *n* = 2 and then *n* = 3 or to use path 2 ($h_{o=1,d=3}=2$) through intersections *n* = 1, *n* = 4 and then *n* = 3 depending on existences of relevant lane markings at various intersections. If all of the required lane markings are available from the optimization process, then one of the two paths or both paths could serve as the input for OD flows.

**Table 1:** Demand OD flows.

(pcu/h)		Destination, *d*				Total flows from origin, *o*
		1	2	3	4	
Origin, *o*	1	-	200	500	400	**1,100**
	2	300	-	200	600	**1,100**
	3	500	300	-	300	**1,100**
	4	400	400	300	-	**1,100**
**Total flows entering destination, *d***		**1,200**	**900**	**1,000**	**1,300**	-

In Fig 7, for some OD pairs, such as from origin *o* = 1 to destination *d* = 2, there are shorter paths that only pass through two intersections to reach their destinations, while the longer paths need to pass through all four intersections in the network. The turning radius is set to be 12.0m for all left- and right-turn movements from both nearside and non-nearside lanes. The arbitrary large positive number *M* takes on 10,000. Clearance times and minimum green duration times are set to be 6.0 and 5.0 seconds, respectively. Effective green duration times are 1.0 seconds longer than the actual (or displayed) green duration times i.e. e=1.0 second. To optimize two green bandwidths (for inbound and outbound traffic in opposite directions) in the present numerical example, traffic flow from origin *o* = 2 to destination *d* = 4 through intersections *n* = 2, *n* = 3, and *n* = 4 is defined as the first selected major path, and another selected major path is from origin *o* = 4 to destination *d* = 2 through the intersections *n* = 4, *n* = 3, and *n* = 2. They are equally important and thus $Z_{o=2,d=4,h_{o,d}=1}=1$ and $Z_{o=4,d=2,h_{o,d}=1}=1$ are set and all other $Z_{o,d,h_{o,d}}$ are set to be zero in the objective function in Eq (32). The distances of all lanes connecting upstream and downstream intersections are assumed to be the same with $\tau_{n,m}=S_{n,m}/{\bar{V}}_{n,m}=26$ seconds.

Based on the sheared delay function, which was calibrated and validated through the NETSIM model using the Hong Kong coordinated network in Sha Tin and their traffic data as inputs, to predict the random-and-oversaturation delays [48] and the Webster’s delay function for calculating the uniform delays (adding them together forming the total delays), the total delays at the ends of links are evaluated using the numerical data results from a signalized network in a previous study [30]. These calculated total delays are set as the target values to be approximated by computing the linearized delay formula in Eq (25). By minimizing the least square error between the two sets of total delays through genetic algorithms, the numerical coefficients of the delay formula in Eq (25) are determined with the following results, $\phi_{n,i,k}=59.25000\cdot \zeta -99.00000\cdot (\Phi_{n,i,k}+e\zeta )+0.39583\cdot y_{n,i,k}-0.00004\cdot \sum_{j}q_{n,i,j,k}$. The predictor variables in Eq (25) are also extracted numerically from the present lane-based network model for predicting the total delays at the ends of lanes.

**Fig 8:**
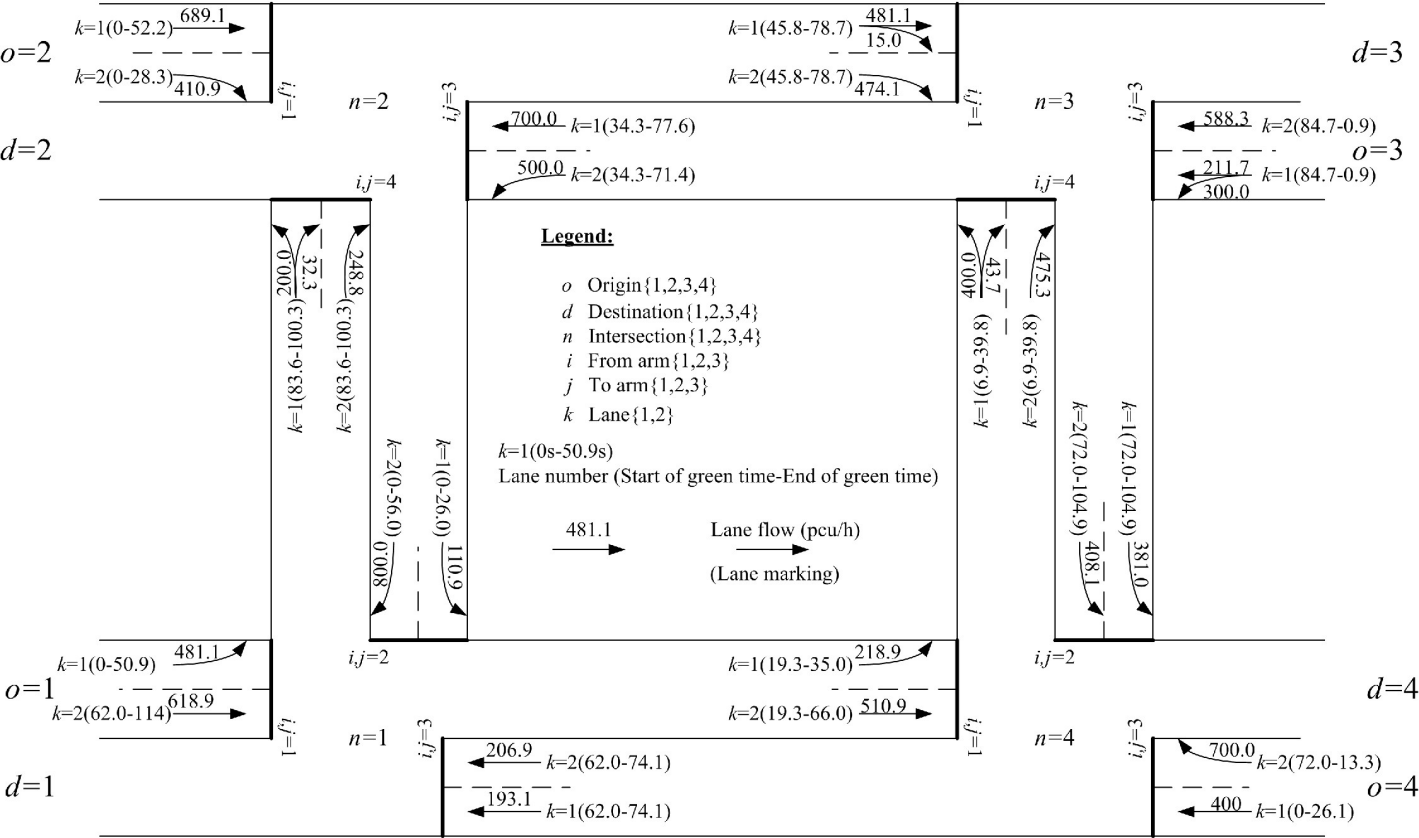
Optimized network configurations including lane markings and assigned lane (turning) flows at individual intersections with ${\bar{W}}_{o=2,d=4,h_{o,d}=1}/\zeta ={\bar{W}}_{o=4,d=2,h_{o,d}=1}/\zeta =32.9s$ (maximized) and ζ=1/120.0.

**Fig 9:**
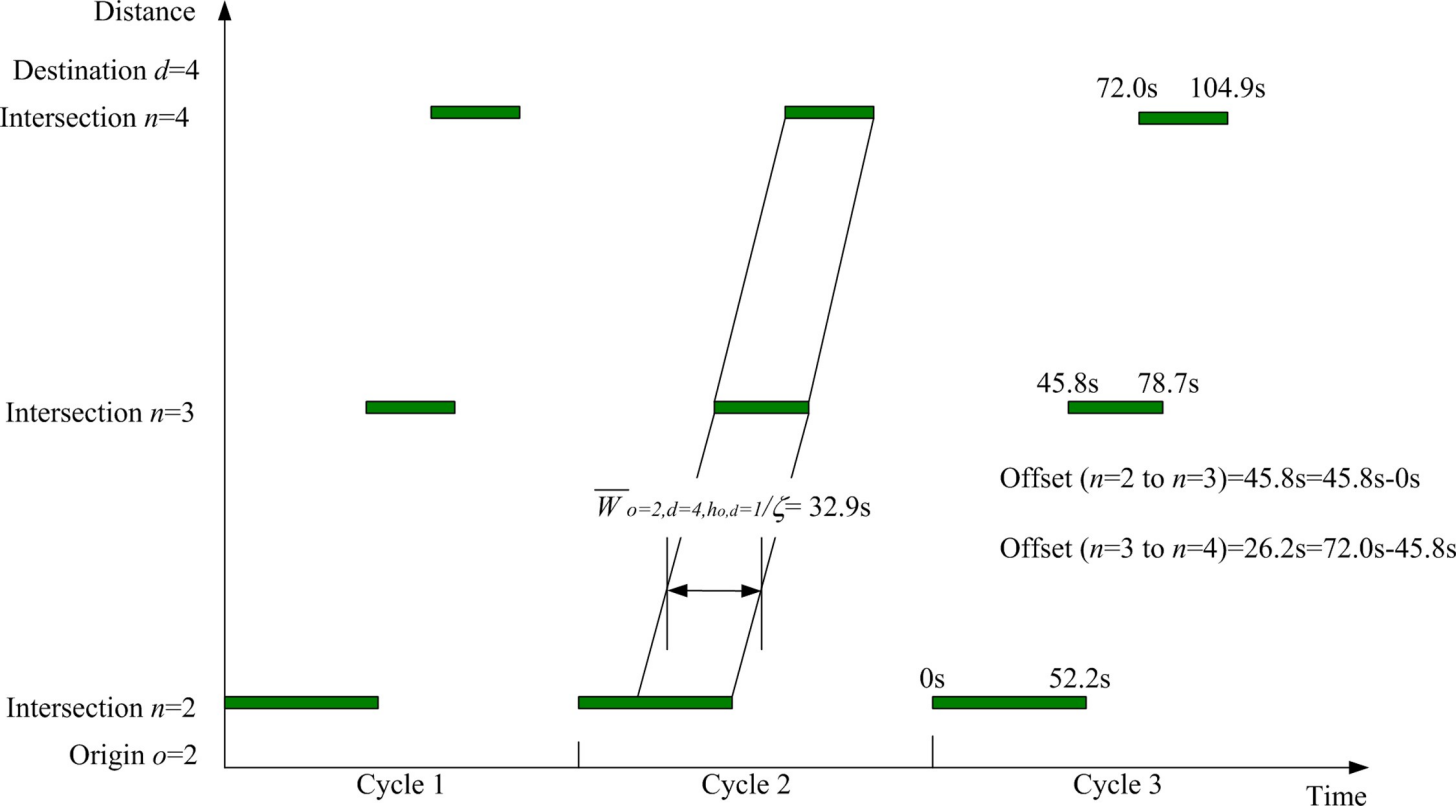
Time-space diagram from origin *o* = 2 at intersection *n* = 2 to destination *d* = 4 at intersection *n* = 4.

**Fig 10:**
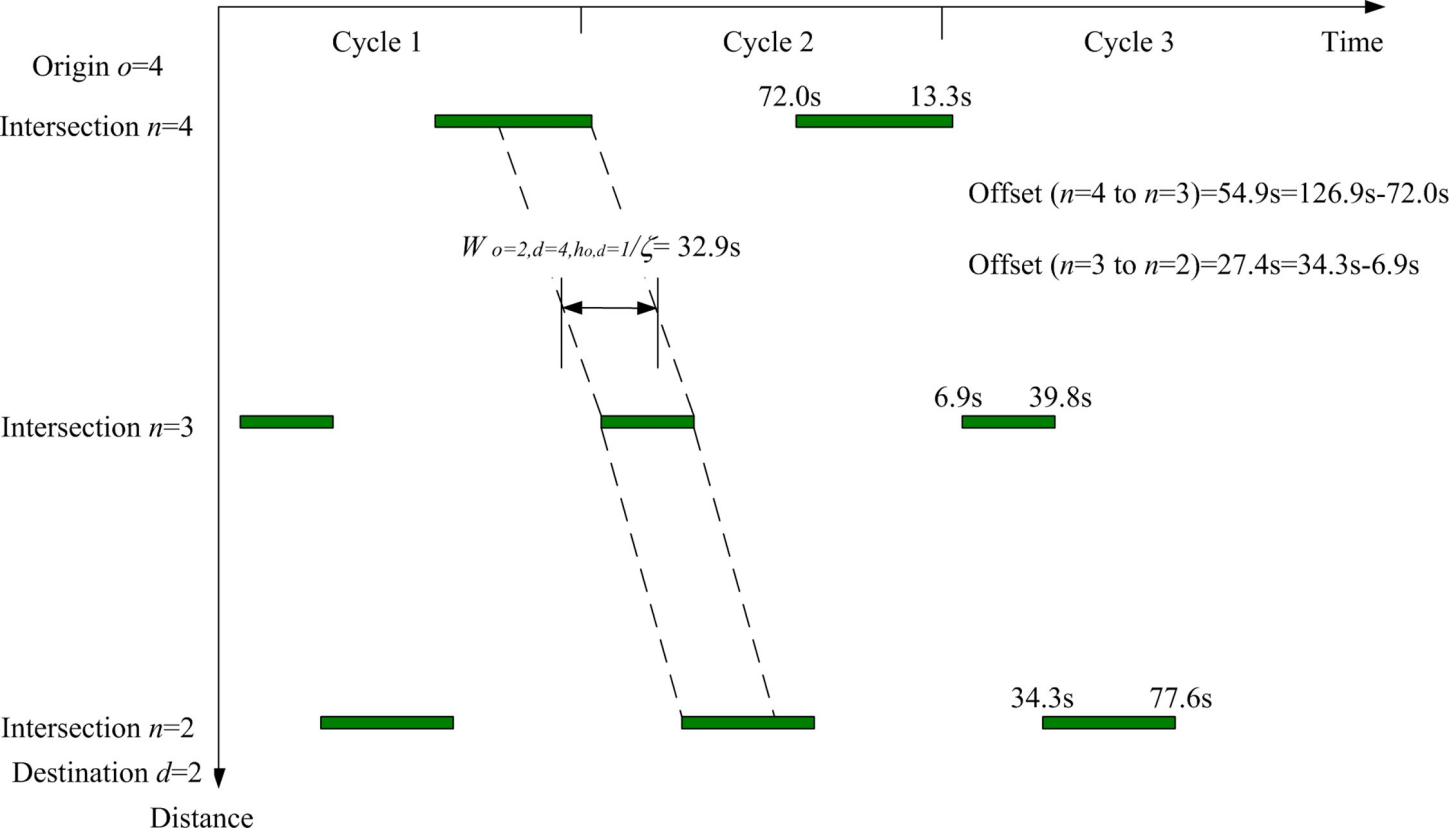
Time-space diagram from origin *o* = 4 at intersection *n* = 4 to destination *d* = 2 at intersection *n* = 2.

Based on all the above model inputs, the proposed lane-based model can be applied to optimize the green bandwidth adopted in objective function Eq (32). Fig 8 shows the details of the optimized lane marking patterns, assigned lane turning flows (in pcu/hr next to the lane markings), green start times, and end of green times (inside brackets) for all modeled lanes and intersections. For the first modeled green band from origin *o* = 2 to destination *d* = 4, intersections *n* = 2, 3, and 4 are visited. Their respective green duration times, forming the continuous green band, are plotted in Fig 9. From the time-space diagram in Fig 9, the optimized green bandwidth ${\bar{W}}_{a=2,b=4,h_{a,b}=1}/\zeta$ for the selected *o* = 2, *d* = 4, and $h_{o,d}$= 1 is 32.9s. Correspondingly, the offsets of the green start times are also modeled to control upstream and downstream traffic. Their offsets are also given in Fig 9. Due to the equal importance from the given numerical weights for the green bands with respect to the inbound and outbound traffic directions, another green band along the opposite traffic direction has the same green bandwidth, ${\bar{W}}_{o=4,d=2,h_{o,d}=1}/\zeta$ = 32.9s as given in Fig 10.

**Fig 11:**
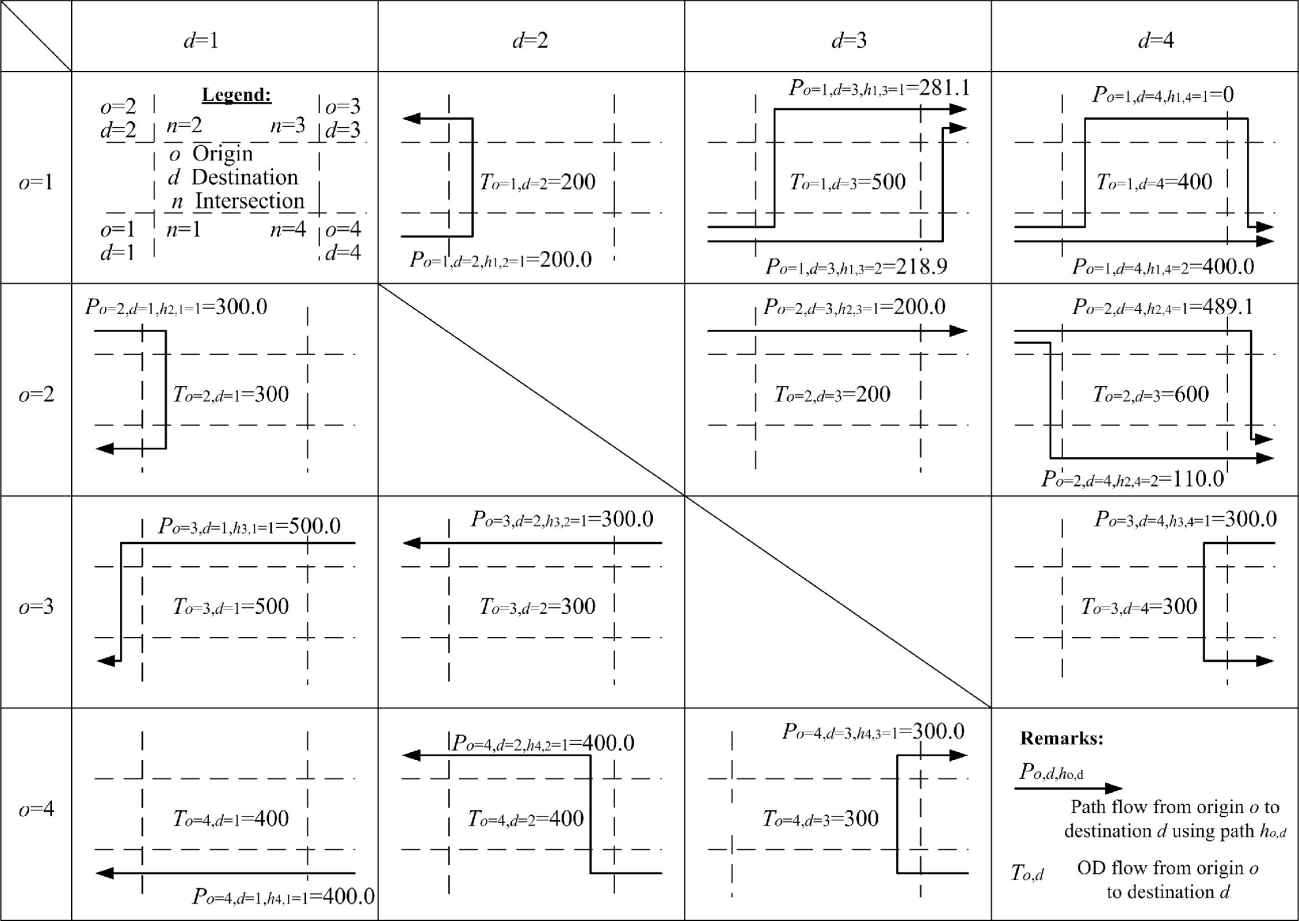
Optimized path flow details for the case study network matching the given OD demand flows (in pcu/h) with ${\bar{W}}_{o=2,d=4,h_{o,d}=1}/\chi ={\bar{W}}_{o=4,d=2,h_{o,d}=1}/\chi =32.9s$ (maximized).

**Fig 12:**
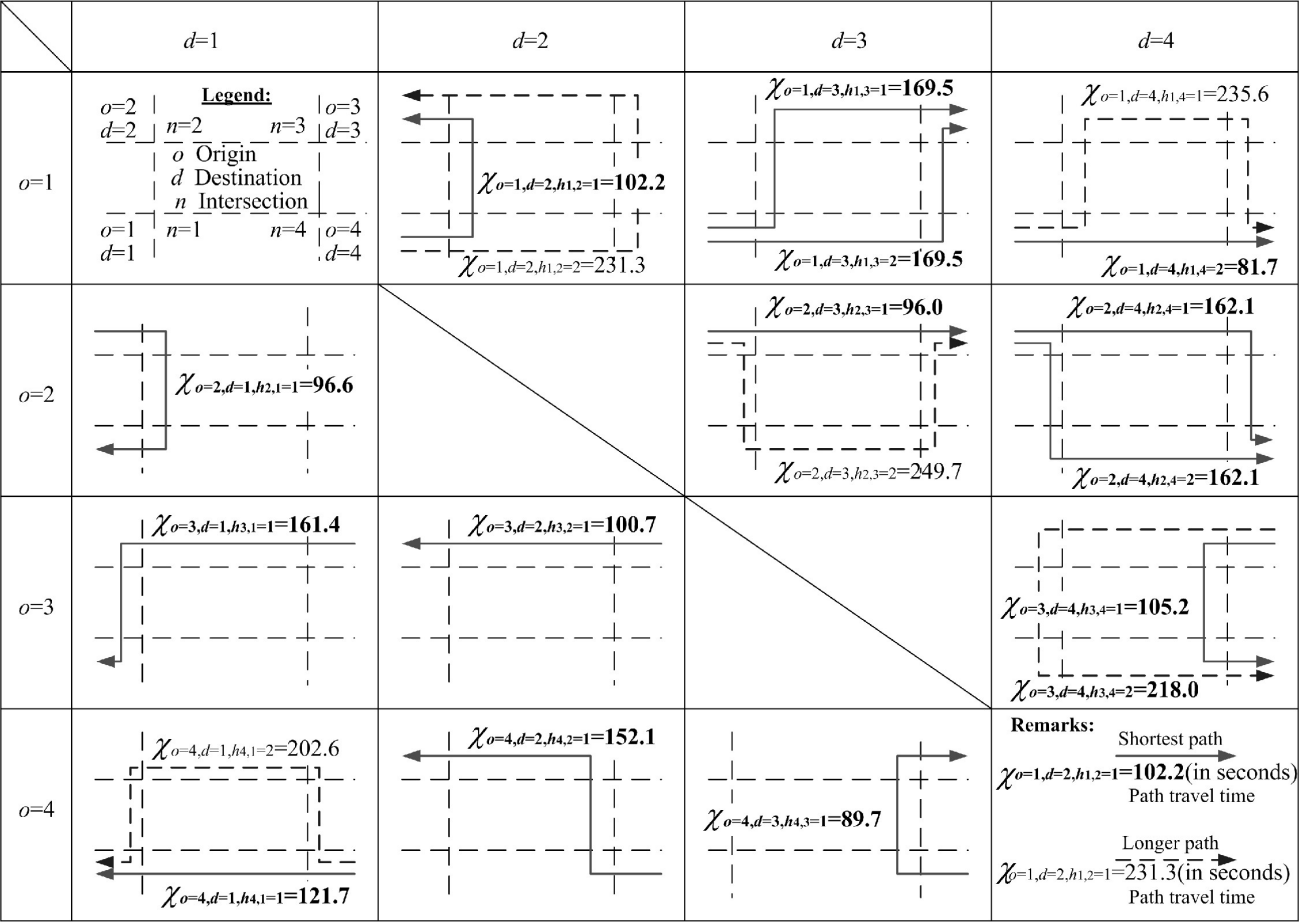
Optimized path travel times for optimizing of green bandwidth with ${\bar{W}}_{a=2,b=4,h_{a,b}=1}/\zeta ={\bar{W}}_{a=4,b=2,h_{a,b}=1}/\zeta =32.9s$ (maximized), μ=1.0 and ζ=1/120.0.

To serve the OD flows $T_{o,d}$ in the example network, different paths are generated and used. Optimized path flows $P(o,d,h_{o,d})$ are obtained and presented in Fig 11. For some OD pairs, two feasible paths are used. And the sum of the path flows equal to the given OD flows due to the flow conservation requirements set in the governing constraints. For example, *o* = 1 and *d* = 3, $T_{o=1,d=3}(=500.0)=P_{o=1,d=3,h_{o,d}=1}(=281.1)+P_{o=1,d=3,h_{o,d}=2}(=218.9)$. Similar results can be found in OD pair *o* = 1, *d* = 4, $T_{o=1,d=3}(=500.0)=P_{o=1,d=4,h_{o,d}=1}(=0.0)+P_{o=1,d=4,h_{o,d}=2}(=400.0)$. It is unusual for the modeled path to show zero path flow. One reason for this is that its corresponding path travel times, as given in Fig 12, which comprise all respective lane cruise times and total delays at the ends of lanes, are $\chi_{o=1,d=4,h_{o,d}=1}=235.6$ seconds and $\chi_{o=1,d=4,h_{o,d}=2}=81.7$ seconds. Due to such path travel time difference, users should choose the shortest path instead of the longer path. Similarly, for OD pair *o* = 1 and *d* = 3, the corresponding path travel times along the two used paths are $\chi_{o=1,d=3,h_{o,d}=1}=\chi_{o=1,d=3,h_{o,d}=2}=169.5$ seconds. Thus, road users from this OD pair could use the two available paths simultaneously.

With the optimized path flows for all OD pairs, flows enter different intersections on different approach lanes following the optimized lane marking patterns at intersections. In the lane-based design framework, these path flows are control inputs for all signalized intersections. Typical model outputs of the path flow distributions onto approach lanes at different intersections are given in Tables 2–5. Generally, total lane flows can be obtained by summing up all turning flows on each lane. If a lane has shared lane marking, two turning flows are allowed in the present example of settings. Associated turning proportions can be determined. Saturation flows could then be revised, taking into consideration turning radius and turning flow proportions. Then, flow factors can be deduced from dividing the total lane flows by the revised saturation flows. These flow factors can also be used to allocate available green duration times to various traffic lanes, and used as model parameters for computing the total delays at ends of lanes using the proposed approximated linear function. Green start and effective green duration times are given in Tables 2–5. The last column presents the degree of saturation for all approach traffic lanes, all of which are unsaturated.

**Table 2:** Optimization results for intersection n = 1 with ${\bar{W}}_{o=2,d=4,h_{o,d}=1}/\zeta ={\bar{W}}_{o=4,d=2,h_{o,d}=1}/\zeta =32.9s$ (maximized), and ζ=1/120.0.

From arm, *i*	Lane, *k*	To arm, *j* Assigned lane flow (pcu/h)			Total lane flow(pcu/h)	Turning proportion	Saturation flow (pcu/h)	Flow factor, $y_{n,i,k}$	Start of green(s)	Effective green(s)	Degree of saturation
		1	2	3							
1	1	-	481.0556	0.0000	481.0556	1.0000	1746.67	0.2754	120.00	51.93	0.64
	2	-	0.0000	618.9444	618.9444	0.0000	2105.00	0.2940	62.00	53.00	0.67
	Total	-	481.0556	618.9444	**1100.0000**						
2	1	0.0000	-	110.8629	110.8629	1.0000	1746.67	0.0635	0.00	27.92	0.27
	2	800.0000	-	0.0000	800.0000	1.0000	1871.11	0.4276	0.00	57.00	0.90
	Total	*800*.*0000*	-	110.8629	910.8629						
3	1	193.1204	0.0000	-	193.1204	0.0000	1965.00	0.0983	62.00	13.11	0.90
	2	206.8796	0.0000	-	206.8796	0.0000	2105.00	0.0983	62.00	13.11	0.90
	Total	*400*.*0000*	0.0000	-	400.0000						

Remarks: Lane marking is prohibited when assigned lane flow = 0.0000 pcu/h; **1100.0000** pcu/h is the total demand flow from origin *o* = 1; *800*.*0000* and *400*.*0000* pcu/h are the total 1200.0000 pcu/h exit flows entering destination *d* = 1.

**Table 3:** Optimization results for intersection n = 2 with ${\bar{W}}_{o=2,d=4,h_{o,d}=1}/\zeta ={\bar{W}}_{o=4,d=2,h_{o,d}=1}/\zeta =32.9s$ (maximized), and ζ=1/120.0.

From arm, *i*	Lane, *k*	To arm, *j* Assigned lane flow (pcu/h)			Total lane flow(pcu/h)	Turning proportion	Saturation flow (pcu/h)	Flow factor, $y_{n,i,k}$	Start of green(s)	Effective green(s)	Degree of saturation
		1	3	4							
1	1	-	689.1371	0.0000	689.1371	0.0000	1965.00	0.3507	120.00	53.15	0.79
	2	-	0.0000	410.8629	410.8629	1.0000	1871.11	0.2196	0.00	29.27	0.90
	Total	-	689.1371	410.8629	**1100.0000**						
3	1	0.0000	-	500.0000	500.0000	1.0000	1746.67	0.2863	34.27	38.16	0.90
	2	700.0000	-	0.0000	700.0000	0.0000	2105.00	0.3325	34.27	44.34	0.90
	Total	*700*.*0000*	-	500.0000	1200.0000						
4	1	200.0000	32.2541	-	232.2541	1.0000	1746.67	0.1330	83.62	17.73	0.90
	2	0.0000	248.8015	-	248.8015	1.0000	1871.11	0.1330	83.62	17.73	0.90
	Total	*200*.*0000*	281.0556	-	481.0556						

Remarks: Lane marking is prohibited when assigned lane flow = 0.0000 pcu/h; **1100.0000** pcu/h is the total demand flow from origin *o* = 2; *700*.*0000* and *200*.*0000* pcu/h are the total 900.0000 pcu/h exit flows entering destination *d* = 2.

**Table 4:** Optimization results for intersection n = 3 with ${\bar{W}}_{o=2,d=4,h_{o,d}=1}/\zeta ={\bar{W}}_{o=4,d=2,h_{o,d}=1}/\zeta =32.9s$ (maximized), and ζ=1/120.0.

From arm, *i*	Lane, *k*	To arm, *j* Assigned lane flow (pcu/h)			Total lane flow(pcu/h)	Turning proportion	Saturation flow (pcu/h)	Flow factor, $y_{n,i,k}$	Start of green(s)	Effective green(s)	Degree of saturation
		1	3	4							
1	1	-	481.0556	14.9990	496.0546	0.0302	1957.60	0.2534	45.82	33.87	0.90
	2	-	0.0000	474.1381	474.1381	1.0000	1871.11	0.2534	45.82	33.87	0.90
	Total	-	*481*.*0556*	489.1371	970.1927						
3	1	211.6861	-	300.0000	511.6861	0.5863	1830.82	0.2795	84.68	37.26	0.90
	2	588.3139	-	0.0000	588.3139	0.0000	2105.00	0.2795	84.68	37.26	0.90
	Total	800.0000	-	300.0000	**1100.0000**						
4	1	400.0000	43.6673	-	443.6673	1.0000	1746.67	0.2540	6.95	33.87	0.90
	2	0.0000	475.2772	-	475.2772	1.0000	1871.11	0.2540	6.95	33.87	0.90
	Total	400.0000	*518*.*9445*	-	918.9445						

Remarks: Lane marking is prohibited when assigned lane flow = 0.000 pcu/h; **1100.0000** pcu/h is the total demand flow from origin *o* = 3; *481*.*0556* and 518.9445 pcu/h are the total 1000.0000 pcu/h exit flows entering destination *d* = 3.

**Table 5:** Optimized results for intersection n = 4 with ${\bar{W}}_{o=2,d=4,h_{o,d}=1}/\zeta ={\bar{W}}_{o=4,d=2,h_{o,d}=1}/\zeta =32.9s$ (maximized), and ζ=1/120.0.

From arm, *i*	Lane, *k*	To arm, *j* Assigned lane flow (pcu/h)			Total lane flow(pcu/h)	Turning proportion	Saturation flow (pcu/h)	Flow factor, $y_{n,i,k}$	Start of green(s)	Effective green(s)	Degree of saturation
		1	2	3							
1	1	-	218.9444	0.0000	218.9444	1.0000	1746.67	0.1253	19.28	16.71	0.90
	2	-	0.0000	510.8629	510.8629	0.0000	2105.00	0.2427	19.28	47.72	0.61
	Total	-	218.9444	*510*.*8629*	729.8073						
2	1	0.0000	-	380.9961	380.9961	1.0000	1746.67	0.2181	72.00	33.87	0.77
	2	0.0000	-	408.1409	408.1409	1.0000	1871.11	0.2181	72.00	33.87	0.77
	Total	0.0000	-	*789*.*1370*	789.1370						
3	1	400.0000	0.0000	-	400.0000	0.0000	1965.00	0.2036	120.00	27.14	0.90
	2	0.0000	700.0000	-	700.0000	1.0000	1871.11	0.3741	72.00	62.28	0.72
	Total	400.0000	700.0000	-	**1100.0000**						

Remarks: Lane marking is prohibited when assigned lane flow = 0.000 pcu/h; **1100.0000** pcu/h is the total demand flow from origin *o* = 4; *510*.*8629* and *789*.*1370* pcu/h are the total 1,300.0000 pcu/h exit flows entering destination *d* = 4.

In one of the equality constraint sets in the formulation, total delays at ends of lanes comprising uniform and random-and-oversaturation delays are used to predict the total delays from origins to destinations across different lanes and intersections. The linearized delay formula in Eq (25) has been proposed to approximate the non-linear uniform and sheared delays. Based on the optimization results, the two delays have been computed and are given in Table 6 for the purposes of comparison and verification. It is found that the two calculated delays for all of the traffic lanes are consistent, with discrepancies found to be within ±2.5%. These findings, which result from using a linear approximation delay formula to replace the non-linear sheared delay function, thus avoiding non-linearity, seem to indicate a possible alternative approach.

**Table 6:** Comparisons of the linear approximated delay and calculated total delay at the end of lane.

Intersection, *n*	Arm, *i*	Lane, *k*	Linear approximated delay (s) in Eq (25), $\varphi_{n,i,k}$	Uniform delay + Sheared delay (s)*, $\phi^{u+s}{}_{n,i,k}$	Error $\frac{\left|\phi^{u+s}{}_{n,i,k}\text{-}\varphi_{n,i,k}\right|}{\phi^{u+s}{}_{n,i,k}}\times 100\text{\%}$
1	1	1	27.0910	26.6475	1.7%
		2	26.4027	26.4988	0.4%
	2	1	38.6795	37.7261	2.5%
		2	28.5278	28.8905	1.3%
	3	1	52.1418	52.8134	1.3%
		2	52.0730	52.8125	1.4%
2	1	1	28.6109	28.6787	0.2%
		2	43.4717	43.9575	1.1%
	3	1	38.8587	39.1040	0.6%
		2	34.9660	35.7371	2.2%
	4	1	49.7780	50.2797	1.0%
		2	49.6953	50.2789	1.1%
3	1	1	40.8652	41.4103	1.3%
		2	40.9748	41.4106	1.1%
	3	1	39.2237	39.5928	0.9%
		2	38.8406	39.5919	1.9%
	4	1	41.1561	41.4450	0.7%
		2	40.9980	41.4445	1.1%
4	1	1	50.3208	50.8413	1.0%
		2	28.8532	28.7485	0.4%
	2	1	39.7651	39.5387	0.6%
		2	39.6294	39.5384	0.2%
	3	1	44.5272	45.1253	1.3%
		2	22.1402	22.1774	0.2%

Remark *: $\phi^{u+s}{}_{n,i,k}$ is the total delay at the end of lane by Webster’s delay and the sheared delay formula in Wong et al. (2003)

## Sensitivity analysis and result comparisons

To illustrate how the proposed algorithms enhance the performance of the optimized network settings, we first optimize the same network by taking the same OD flow matrix and other network settings as inputs, specifically by maximizing the common flow multiplier according to the method developed from a recent study [31] and compare their results. In addition, to conduct a sensitivity analysis, all shared lane markings in the optimized network configuration are removed for evaluation. Five performance indicators are retrieved from the optimization results for comparison: (I) the length of green bands along major paths, (II) the numbers and patterns of lane marking arrows on approach traffic lanes to enable feasible paths serving the given OD flows, (III) the effectiveness of signal timing for coordinating upstream and downstream traffic signals, (IV) existence of equilibrium flow conditions for using different paths to connect the same OD pairs, and (V) total network travel times $\left(=\sum_{o=1}^{O}\sum_{d=1}^{D}\sum_{h_{o,d}=1}^{{\bar{H}}_{o,d}}P_{o,d,h_{o,d}}\cdot \chi_{o,d,h_{o,d}}\right)$. The methods and results chosen for comparison are obtained from the latest method used to establish network link connections by varying and optimizing lane markings at the intersection levels. The method maximizes the common flow multiplier of the given OD flow matrix. The scaled OD flows that enter the study network are subject to a simple requirement for flow conservation based on equalizing the given OD flows and the sum of path flows in the used paths. The method optimizes the traffic signal settings for all individual intersections without modeling traffic signal coordination, which is expected to be refined by the proposed study method. The proposed method enhances the five performance indicators mentioned above, allowing them to obtain more convincing and realistic results.

**Fig 13:**
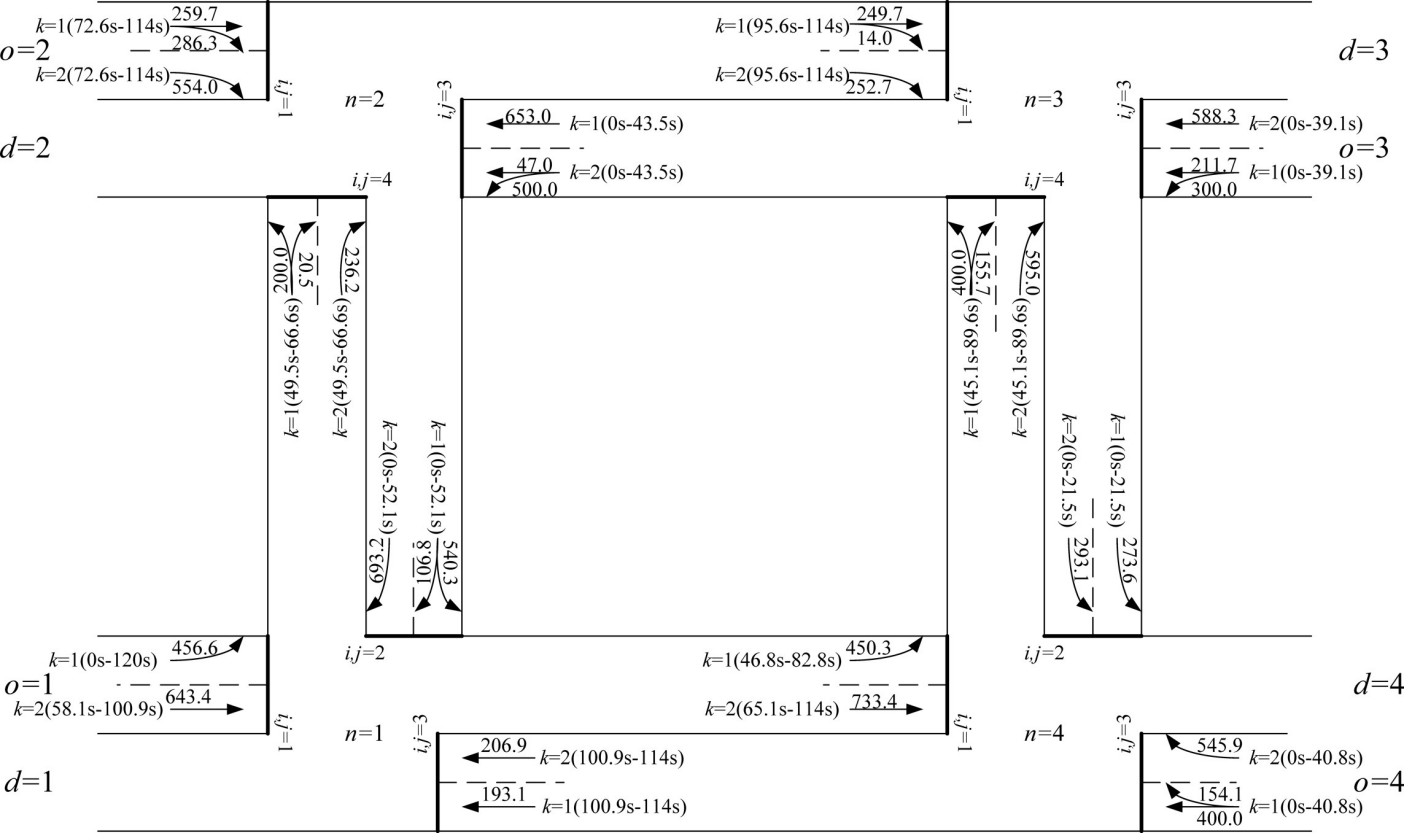
The optimized network configurations by maximizing the common flow multiplier μ (= 1.075) with cycle length ζ=1/120.0s.

For performance indicator (I) the widths of green bands (across 3 intersections), the previous method give 15.4 s for connecting origin *o* = 2 to destination *d* = 4 through intersection *n* = 3 and zero in the opposite direction. It also records 2.1s for connecting origin *o* = 3 to destination *d* = 1 through intersection *n* = 2 and zero in its opposite direction, according to the green start and duration times given in Fig 13. The proposed algorithms are applied to two major paths defined by users as inputs connecting origin *o* = 2 to destination *d* = 4 through intersection *n* = 3 (inbound direction) and its opposite direction connecting origin *o* = 4 to destination *d* = 2 through intersection *n* = 3 (outbound direction). The sum of the OD flows for this OD pair is the largest (= 600 + 400 = 1,100 pcu/h). The proposed MAXBAND optimization method gives 32.9 seconds of green bandwidths for both inbound and outbound traffic directions, as plotted in Figs 9 and 10. Total green bandwidth is 65.8 seconds, as optimized by the proposed algorithms, while 17.5 seconds (total green bandwidth = 15.4 + 2.1) is found by the previous method.

For performance indicator (II), the proposed algorithms optimize 28 lane marking arrows in the study network, as shown in Fig 8, while the previous method optimize 32 lane marking arrows, as shown in Fig 13. Both settings can connect the four OD pairs with 15 modeled paths. It can thus be concluded that the proposed algorithms rendered the optimized lane marking patterns more efficiently.

For performance indicator (III), the proposed algorithm’s optimized signal timings are more effective in generating longer green bands along users’ specified major paths, as found in performance indicator (I). Both sets of signal timing results, as given in Figs 8 and 13, are able to separate conflicting traffic movements at the intersection level. Hence, the proposed optimization algorithms lead to better coordination of optimized traffic signal settings.

**Fig 14:**
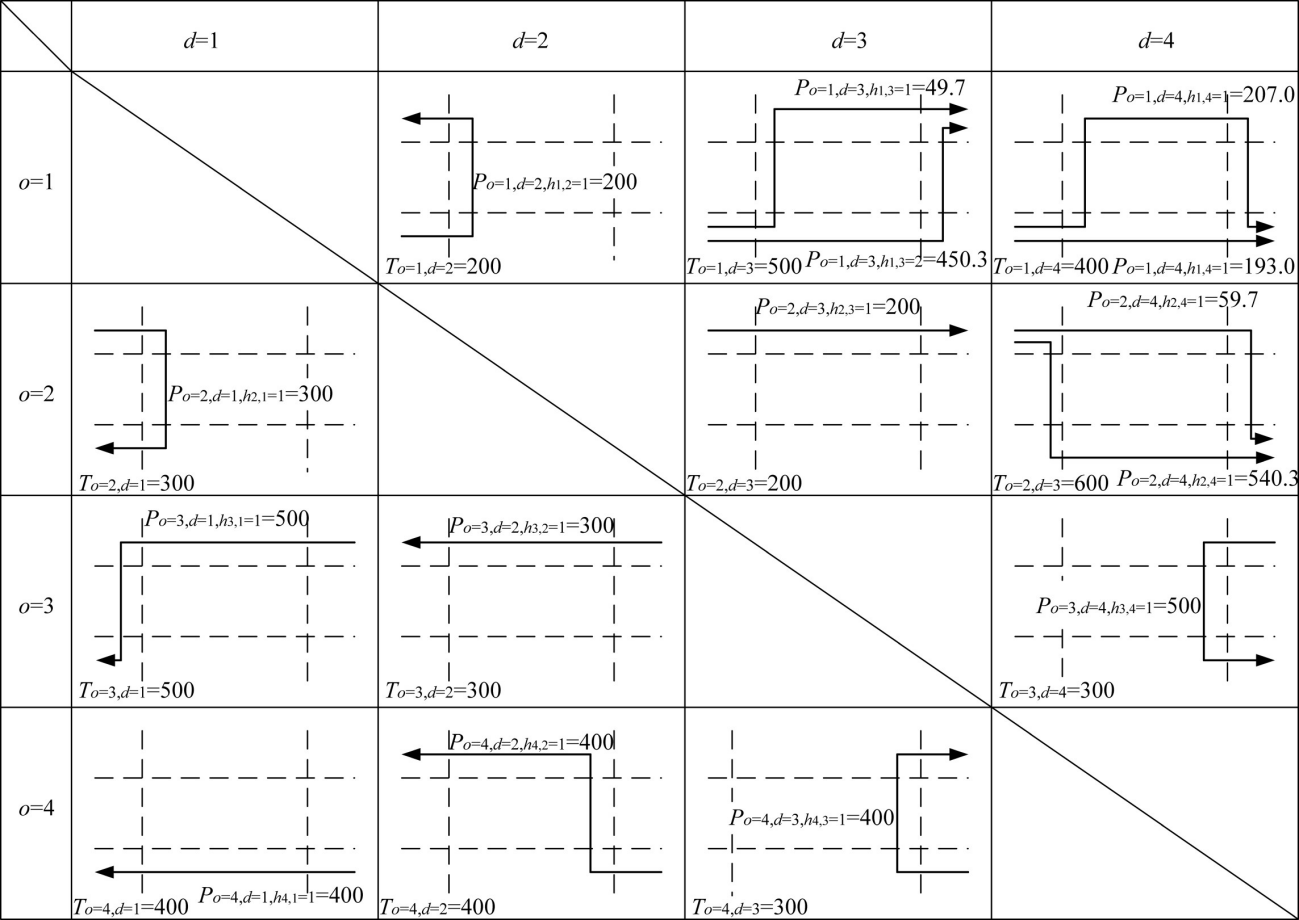
Path flow patterns by maximizing the common flow multiplier μ (= 1.075) with cycle length ζ=1/120.0s.

As for performance indicator (IV), users from the same OD pair, under equilibrium conditions, may travel using different paths and no user should be able to save travel time by switching to another path. In addition, the resultant path travel times for the same OD pairs should be identical. The proposed algorithms develop the necessary governing constraint sets to control path flows, satisfying this path travel time requirement. From origin *o* = 1 to destination *d* = 3 or 4 and *o* = 2 to *d* = 4, there are two available paths optimized by the previous method, as given in Fig 14 and Table 7. Their respective path travel times are not identical, violating the equilibrium requirement. This problem is completely resolved by the proposed design algorithms presented in Fig 12 and Table 8. The two path travel times are now equalized: the path from *o* = 1 to *d* = 3 is now equalized to 169.5s and the path from *o* = 2 to *d* = 4 is now equalized to 162.1s. For path 1 and path 2, path travel times from *o* = 1 to *d* = 4 are now 235.6 seconds and 81.7 seconds, respectively. The OD flow (= 400 pcu/h) uses path 2 with a shorter travel time (= 81.7s) and the flow along path 1 is zero due to a longer travel time (= 235.6s).

**Table 7:** Details of path flows and path travel times by maximizing the common flow multiplier μ (= 1.075) with cycle length ζ=1/120.0s.

Origin, *o*	Destination, *d*	Path, $h_{o,d}$	Path flow, $P_{o,d,h_{o,d}}$ (pcu/h)	Path travel time, $\chi_{o,d,h_{o,d}}$ (s)	Total path travel time, $P_{o,d,h_{o,d}}$x $\chi_{o,d,h_{o,d}}$ (pcu-h/h)
1	2	1	200.0	75.2	4.2
	3	1	49.7	149.5	2.1
		2	450.3	159.0	19.9
	4	1	207.0	222.3	12.8
		2	193.0	91.3	4.9
2	1	1	300.0	91.1	7.6
	3	1	200.0	110.0	6.1
	4	1	59.7	182.8	3.0
		2	540.3	148.2	22.2
3	1	1	500.0	152.7	21.2
	2	1	300.0	96.5	8.0
	4	1	300.0	109.7	9.1
4	1	1	400.0	113.1	12.6
	2	1	400.0	155.8	17.3
	3	1	300.0	95.7	8.0
					Total = 159.0

**Table 8:** Details of path flows and path travel times by the proposed algorithms.

Origin, *o*	Destination, *d*	Path, $h_{o,d}$	Path flow, $P_{o,d,h_{o,d}}$ (pcu/h)	Path travel time, $\chi_{o,d,h_{o,d}}$ (s)	Total path travel time, $P_{o,d,h_{o,d}}$x $\chi_{o,d,h_{o,d}}$ (pcu-h/h)
1	2	1	200.0	102.2	4.2
	3	1	281.1	169.5	2.1
		2	218.9	169.5	19.9
	4	1	0.0	235.6	12.8
		2	400.0	81.7	4.9
2	1	1	300.0	96.6	7.6
	3	1	200.0	96.0	6.1
	4	1	489.1	162.1	3.0
		2	110.9	162.1	22.2
3	1	1	500.0	161.4	21.2
	2	1	300.0	100.7	8.0
	4	1	300.0	105.2	9.1
4	1	1	400.0	121.7	12.6
	2	1	400.0	152.1	17.3
	3	1	300.0	89.7	8.0
					Total = 156.2

For performance indicator (V), both methods model 15 paths in the network, using them to serve the four OD pairs shown in Figs 11 and 14. By comparing the 15 path travel times optimized by the proposed algorithms, given in Fig 12 and Table 8, with those optimized by the previous method, given in Table 7, the algorithms reduce the total network travel times of all modeled paths (= 156.2 pcu-h/h in Table 8) more than the previous method (= 159.0 pcu-h/h in Table 7).

With respect to the optimized network configuration shown in Fig 8, the four shared lane markings demonstrate that the proposed lane-based optimization method is able to use all approach lanes effectively and optimize the lane turning patterns by fixing proper shared lane locations. In practical designs, engineers and network planners may design simple network configurations without involving shared lane markings. A sensitivity analysis compares the five performance indicators with respect to the optimized network configuration in Fig 8 and the simple network design (by removing the four shared lane markings from the optimized network configuration). The summary of those comparisons is given in Table 9. The proposed lane-based optimization method, which optimizes the network configuration with shared lane marking designs, outperforms the simple network design.

**Table 9:** Summary of sensitivity analysis comparing the optimized network settings and a simple network design without shared lane markings.

Scientific performance indicators	Optimized design	Simple design without shared lane markings
(I) Lengths of green bands	32.9 seconds^^^	31.2 seconds
(II) Numbers and patterns of lane markings to enable feasible paths for connecting OD pairs	28^#^ (with four optimized lane markings)	24 (without shared lane markings)
(III) Effectiveness of signal timings to coordinate upstream and downstream intersections	Degree of saturation for all approach traffic lanes are below 0.9 (without overflowing)	Degree of saturation on two approach traffic lanes *k* = 1 and 2 at intersection *n* = 2 and arm *i* = 4 is over 0.9 (overflowing). Their degrees of saturation are 2.29 and 5.21, respectively*.
(IV) Existence of equilibrium flow conditions for using different paths to connect the OD pairs	YES (and without overflowing found in all approach lanes)	YES (but two approach lanes are overflowing)
(V) Total network travel times	156.2 pcu-h/h	161.2 pcu-h/h

Remarks:

^ Lengths of green bands are longer in the optimized network settings

# Four optimized shared lane markings are given in the optimized network settings

* Degree of saturation is below 0.9 for all approach traffic lanes in the optimized network settings

## Conclusions

This study’s main innovations are the extension of the lane-based design concept to the optimization of individual lane usage, which includes lane markings, assigned lane flows, and the traffic signal settings used to configure a signalized network. The lane markings at individual intersections are defined as binary type variables and new governing constraint sets are developed them as network links. Available paths can then be established to serve the users’ input OD flows. Efficient network link connections can obtained if proper lane markings are optimized. Multiple paths can also be generated to connect the origin and destination pairs through different turns at different intersections, according to the optimized lane marking patterns. Link connectivity and network configuration are enhanced and optimized in a unified framework that combines assigned lane flows and traffic signal settings. The proposed bandwidth optimization method facilitates the coordination of the traffic signal settings that control individual signal-controlled intersections. The offsets in regulating green start times across upstream and downstream intersections are also optimized. All of these are new features for extending the lane-based method to the design of signalized networks. The problem is formulated as a BMILP that can solved by a standard branch-and-bound routine. A numerical example with four intersections in a network with two approach lanes and two exit lanes is given to demonstrate the effectiveness of the proposed algorithms. Five performance indicators have been extracted from the optimization results and show that the proposed algorithms outperform the previous method that was developed to optimize the common flow multiplier, but without considering the bandwidth optimization for traffic signal coordination. To summarize, the optimized bandwidth is found to be 32.9 seconds and the total network travel time is 156.2 pcu-h/h. Future research on network configuration designs should incorporate safety performance to guard against shared lane markings on non-nearside lanes permitting three movement turns that may lead to statistically higher accident rates. In designing large-size networks, such restrictions should be imposed. The potential tradeoffs between engineering performance and safety considerations also merit future research attention.
